# Municipal wastewater monitoring revealed the predominance of *bla*_GES_ genes with diverse variants among carbapenemase-producing organisms: high occurrence and persistence of *Aeromonas caviae* harboring the new *bla*_GES_ variant *bla*_GES-48_

**DOI:** 10.1128/spectrum.02188-23

**Published:** 2023-10-09

**Authors:** Mizuki Tanabe, Yo Sugawara, Tomohiro Denda, Kanae Sakaguchi, Shino Takizawa, Shota Koide, Wataru Hayashi, Liansheng Yu, Shizuo Kayama, Motoyuki Sugai, Yukiko Nagano, Noriyuki Nagano

**Affiliations:** 1 Department of Health and Medical Sciences, Graduate School of Medicine, Shinshu University, Matsumoto, Nagano, Japan; 2 Antimicrobial Resistance Research Center, National Institute of Infectious Diseases, Higashimurayama, Tokyo, Japan; 3 Department of Medical Sciences, Shinshu University, Graduate School of Medicine, Science and Technology, Matsumoto, Nagano, Japan; JMI Laboratories, North Liberty, Iowa, USA

**Keywords:** carbapenemase, wastewater, *bla*_GESs_, *bla*_IMPs_, *Aeromonas *spp., *Enterobacterales*, class 1 integron

## Abstract

We characterized carbapenemase-producing organism (CPO) detected in municipal wastewater to better understand the epidemiology of CPOs in the community. In total, 36 samples were collected at six sampling sites every other month from December 2020 to October 2021. CPOs were not recovered from influent taken from inlet A receiving separated sewer line, treated effluents, and river water samples upstream and downstream of the effluent outlet. By contrast, 75 CPOs were detected in all influent samples taken from inlets B and C receiving combined sewer lines collecting both domestic/industrial wastewater and rainwater runoff. *Aeromonas caviae* was the dominant species (25/75, 33.3%), and the other 11 *Aeromonas* spp. together accounted for 48% of CPOs. The remaining 39 *Enterobacterales* strains mainly comprised 17 *Klebsiella* spp. and 10 *Raoultella* spp. CPOs carrying *bla*_GES_ carbapenemase genes were overwhelmingly dominant, accounting for 72 of 75 isolates, including two isolates harboring both *bla*_GES-24_ and *bla*_IMP-1_ (96%), followed by three *bla*_IMPs_-positive isolates, where those carbapenemase genes were mainly carried in diverse class 1 integrons. Among *bla*_GES_ variants, including six new variants (*bla*_GES-47_, *bla*_GES-48_, *bla*_GES-49_, *bla*_GES-50_, *bla*_GES-51_, and *bla*_GES-54_), *bla*_GES-5_ was detected in 28 CPOs, with *Aeromonas* spp. accounting for 53.6% of these organisms. Quantitative analysis revealed that the repeated detection of *bla*_GES-48_-positive *A. caviae* ST1056 from both inlets B and C ranked the total number of this bacterial clone highest in the wastewater influent. In summary, our study revealed the high prevalence and persistence of diverse *bla*_GES_ carbapenemase genes among CPOs isolated from influent inlets connected to combined sewer systems.

**IMPORTANCE:**

The emergence and spread of carbapenemase-producing organisms (CPOs) represent a global health threat because they are associated with limited treatment options and poor clinical outcomes. Wastewater is considered a hotspot for the evolution and dissemination of antimicrobial resistance. Thus, analyses of municipal wastewater are critical for understanding the circulation of these CPOs and carbapenemase genes in local communities, which remains scarcely known in Japan. This study resulted in several key observations: (i) the vast majority of *bla*_GES_ genes, including six new *bla*_GES_ variants, and less frequent *bla*_IMP_ genes were carbapenemase genes encountered exclusively in wastewater influent; (ii) the most dominant CPO species were *Aeromonas* spp., in which a remarkable diversity of new sequence types was observed; and (iii) CPOs were detected from combined sewer wastewater, but not from separate sewer wastewater, suggesting that the load of CPOs from unrecognized environmental sources could greatly contribute to their detection in influent wastewater.

## INTRODUCTION

Antimicrobial resistance (AMR) represents a major global problem linked to increasing concerns across human, animal, and environmental settings. Carbapenems remain the last-line antimicrobials for treating multidrug-resistant microorganism infections. Thus, the emergence and spread of carbapenemase-producing organisms (CPOs) represent a global health threat because they are associated with limited treatment options and poor clinical outcomes. Clinically important carbapenemases belonging to different Ambler classes include class A carbapenemase *Klebsiella pneumoniae* carbapenemase (KPC) type, class B metallo-β-lactamases IMP-type metallo-β-lactamase (IMP) type, Verona integron-encoded metallo-β-lactamase (VIM) type, and New Delhi metallo-β-lactamase (NDM) type, and class D carbapenemase OXA-48 like. Among the class A β-lactamases of the Guiana extended spectrum β-lactamase (GES) type, those with an amino acid substitution of Gly170Ser within the Ω-loop region, such as GES-24 and GES-5, exhibit carbapenem-hydrolyzing activities. The relatively low carbapenem minimum inhibitory concentrations (MICs) of some isolates of GES carbapenemase producers in combination with the lack of available selective inhibitors specific for GES carbapenemases may make it difficult to detect these enzymes, leading to an underestimation of the true prevalence of GES carbapenemase-producing isolates. These carbapenemase genes are often located in mobile genetic elements (MGEs) such as plasmids, transposons, and integrons, thereby facilitating their rapid spread among *Enterobacterales* and other Gram-negative bacteria (1). The acquisition of MGEs by high-risk bacterial clones with adaptive traits in humans and environments with accumulating virulence and resistance genes plays critical roles in the successful dissemination of carbapenemase genes (2). The geographic distribution of CPOs is variable. Generally, the high endemicity of certain carbapenemases is associated with specific regions or countries, such as the KPC type in the USA, Israel, Greece, and Italy; NDM type in the Indian subcontinent; OXA-48 like in Turkey, the Middle East, and North Africa; and IMP type in East Asian countries including Japan (3, 4). Moreover, several factors, such as international travel/migration, repatriation of patients, food import, and wildlife migration from areas of high endemicity, can accelerate the extensive spread of CPOs to neighboring regions, surrounding countries, and other continents (5 – 8), leading to constant changes in local and global epidemiology.

In Japan, the prevalence of carbapenemase-producing *Enterobacterales* among carbapenem-resistant *Enterobacterales* (CRE) clinical isolates remained flat at 16.5%–17.6% in 2018–2020 [Pathogen surveillance of carbapenem-resistant *Enterobacteriaceae*, 2020 (In Japanese). Infect. Agents Sruveillance Rep. 2022, 43, 215–216, available online at https://www.niid.go.jp/niid/ja/cre-m/cre-iasrd/11520-511d01.html]. Japan has been characterized as endemic for the IMP type (approximately 85% of isolates), whereas the NDM type, KPC type, and OXA-48 like are sporadically detected. Among IMP-type enzymes, IMP-1 is the most prevalent, whereas IMP-6 producers, which originally exhibited a regional-specific distribution while causing large-scale outbreaks, have expanded their areas of distribution across Japan (9, 10). With increasing concerns about CPO spread, regional surveillance is essential to understand their epidemiology.

Analyses of wastewater containing bacteria mainly of human origin could be helpful for better understanding the community prevalence of antimicrobial-resistant bacteria, AMR genes, and associated MGEs (11). Recent studies revealed the presence of carbapenemase-encoding genes, such as *bla*_KPC_, *bla*_NDM_, *bla*_IMP_, and *bla*_OXA-48_ in wastewater influent in different countries, and the concordance of their prevalence in wastewater and human isolates was generally demonstrated (12 – 14). Meanwhile, the predominance of *bla*_GES_ carbapenemase genes mainly harbored by *Aeromonas*, *Enterobacter*, and *Kluyvera* spp. in wastewater influent has been reported in Finland, where *bla*_GES_ has rarely been detected in clinical isolates (15). GES carbapenemases are relatively rare among clinical isolates, whereas they are increasingly detected in aquatic environments (16). Nonetheless, outbreaks of GES-5-producing *Pseudomonas aeruginosa* and *Serratia marcescens* have been reported in Japan (17, 18). In a very recent study, we found that *bla*_GES-24_ and *bla*_GES-5_, which are mainly carried by *Klebsiella* and *Enterobacter* spp., are the only carbapenemase genes detected in wastewater effluent from a hospital with no history of detection of clinical GES carbapenemase-producing isolates (19). Notably, these *bla*_GES_ genes were mostly embedded in new class 3 integrons, although they are generally associated with class 1 integrons in clinically relevant *Enterobacterales* (19, 20). These findings prompted us to investigate the epidemiology of CPOs in local communities.

In this study, quantitative monitoring of CPOs in influent from a municipal wastewater treatment plant (WWTP) was conducted to investigate their presence, distribution, and dynamics. This WWTP receives sewer water in a combined sewer system that primarily collects domestic/industrial wastewater mixed with urban runoff rainwater, as well as in the separated sewer system that carries only domestic/industrial wastewater via different inlets. Therefore, the influent of these inlets was analyzed to assess the environmental impact of the CPO load from the combined sewer system. Additionally, wastewater effluent and river water samples (upstream and downstream of the effluent outlet) were also included in the analysis to better understand whether the effluent is a potential source of CPO release into the environment.

## RESULTS

### Detection of CPOs in the influent and effluent of a WWTP and river water samples

In total, 36 samples were collected at six sampling sites once every 2 months between December 2020 and October 2021. CPOs were detected in all six raw influent samples taken from inlets B (pH, 7.3 ± 0.1; temperature, 18.3 ± 3.0) and C (pH, 7.3 ± 0.1; temperature, 19.5 ± 3.7) receiving combined sewer lines. No CPOs were recovered from the raw influent of inlet A (pH, 7.4 ± 0.1; temperature, 18.5 ± 3.4) receiving separated sewer lines, treated effluent (pH, 7.0 ± 0.2; temperature, 19.5 ± 3.6) from the WWTP outlet, and river water samples (upstream: pH, 7.0 ± 0.8; temperature, 13.3 ± 3.5; downstream: pH, 6.9 ± 0.7; temperature, 13.8 ± 3.7; Table S1).

A total of 75 CPOs were selected as representative strains of bacterial populations, estimated as colony-forming units (CFU) per milliliter, sharing similar colony morphologies, the same bacterial species, and the same carbapenemase genes. Table 1 shows bacterial species based on average nucleotide identity based on MUMmer (ANIm) analyses, carbapenemase genes, sequence types (STs), and strain numbers for 75 CPOs identified in the raw influent of inlets B (28 isolates) and C (47 isolates). *Aeromonas caviae* was the dominant species (25/75, 33.3%), and 11 other *Aeromonas* spp. (four *Aeromonas veronii* bv. *veronii* isolates, three *Aeromonas hydrophila* subsp. *hydrophila* isolates, two *Aeromonas taiwanensis* isolates, and one isolate each of *Aeromonas dhakensis* and *Aeromonas allosaccharophila*) collectively accounted for 48% of CPOs. Other isolates included seven isolates each of *Klebsiella michiganensis* and *Raoultella ornithinolytica*; five isolates each of *Klebsiella pneumoniae* subsp. *pneumoniae* and *Klebsiella quasipneumoniae* subsp. *similipneumoniae*; three isolates each of *Citrobacter freundii* and *Raoultella planticola*; two isolates each of *Enterobacter cloacae* subsp. *cloacae*, *Enterobacter kobei*, and *Enterobacter roggenkampii*; and one isolate each of *Citrobacter braakii*, *Citrobacter portucalensis*, and *Kluyvera cryocrescens*.

**TABLE 1:** Seventy-five strains harboring carbapenemase genes identified in raw influents of inlets B and C^*a*^

Sampling date (month/year)						Dec-20	Feb-21	Apr-21	Jun-21	Aug-21	Oct-21	Dec-20	Feb-21	Apr-21	Jun-21	Aug-21	Oct-21
Inlet						B						C					
Water temperature						17°C	13°C	17°C	20°C	22°C	21°C	18°C	13°C	18°C	21°C	24°C	23°C
pH						7.47	7.41	7.29	7.36	7.37	7.06	7.53	7.29	7.28	7.09	7.19	7.17
Bacterial species	Integron	Carbapenemases	Others	ST^*b*^	*Klebsiella* capsule locus (KL) type	Strain (single nucleotide polymorphisms [SNPs])^*d*^						Strain (single nucleotide polymorphisms [SNPs])^*d*^					
*A. allosaccharophila*	3	GES-24	TEM-1B	1,180^*c*^													10CC9
*A. caviae*	1	IMP-10		1,061^*c*^					6BC7								
	3	GES-5		1,015^*c*^		12BC1				8BC4	10BC1						
										(365)	(19)						
	1	GES-5		1,057^*c*^										4CC9			
	1	GES-5	MOX + VEB-1	1,059^*c*^							10BC8		2CC7	4CC12			
											(31)			(19)			
	1	GES-5	MOX + VEB-1	1,176^*c*^													10CC10
	1	GES-5	MOX	1,178^*c*^					6BC8						6CC6		
															(59)		
	3	GES-6		1,182^*c*^											6CC8		
	1	GES-24		1,199^*c*^					6BC6								
	1	GES-24		1,179^*c*^							10BC6						
	1	GES-24 + IMP-1		1,062^*c*^											6CC5		
	1	GES-48*^a^*		1,056^*c*^			2BC3	4BC2	6BC4	8BC3	10BC4		2CC8	4CC5	6CC12		10CC8
								(274)	(9)	(63)	(211)		(63)	(60)	(9)		(8)
	1	GES-48*^a^*		1,182^*c*^												8CC4	
*A. dhakensis*	1	GES-24		518													10CC4
*A. hydrophila* subsp. *hydrophila*	1	GES-5		1,181^*c*^											6CC13		
	1	GES-24		1,177^*c*^												8CC6	
	1	GES-24 + IMP-1		860^*c*^												8CC5	
*A. taiwanensis*	3	GES-5		1,055^*c*^			2BC2	4BC3									
								(835)									
*A. veronii* bv. *veronii*	1	GES-5		1,054^*c*^		12BC2	2BC1										
							(30)										
	1	GES-24		1,058^*c*^									2CC6				
	1	GES-49*^a^*		1,060^*c*^					6BC9								
*C. braakii*	1	GES-5		110						8BC2							
*C. portucalensis*	1	GES-51*^a^*		166													10CC7
*C. freundii*	1	GES-24	CTX-M-3 + TEM-1B	22										4CC11			
	1	IMP-1	CTX-M-3 + TEM-1B	116								12CC1	2CC2				
													(12)				
*E. cloacae* subsp. *cloacae*	1	GES-4		1,821^*c*^						8BC1	10BC2						
											(19)						
*E. kobei*	1	GES-4		1,822^*c*^											6CC1		
	1	GES-54*^a^*		520					6BC5								
*E. roggenkampii*	1	GES-4		1,059										4CC4			
	1	GES-24	MCR-9	433					6BC1								
*K. cryocrescens*	1	GES-6		−—					6BC3								
*K. michiganensis*	1	GES-4		95								12CC2		4CC2	6CC2	8CC1	
														(15)	(13)	(24)	
	1	GES-47*^a^*		135									2CC1				
	1	GES-50*^a^*		210			2BC4				10BC5						
											(65)						
*K. pneumoniae* subsp. *pneumoniae*	1	GES-5		2,791	KL48				6BC2					4CC3	6CC4		10CC2
									(100)						(138)		(74)
	1	GES-48*^a^*		76	KL35												10CC1
*K. quasipneumoniae* subsp. *similipneumoniae*	1	GES-4		6,101^*c*^	KL159										6CC9		
	1	GES-5		1,822	KL48									4CC10	6CC7		10CC6
															(30)		(59)
	1	GES-48*^a^*		6,102^*c*^	KL140											8CC2	
*R. planticola*	1	GES-5		–								12CC3	2CC3	4CC1			
													(115)	(116)			
*R. ornithinolytica*	1	GES-4		–										4CC7	6CC3	8CC3	10CC5
															(111)	(7)	(30)
	1	GES-5		–									2CC4		6CC10		
															(66)		
	1	GES-24		–				4BC1									

^*a*^ GES variants newly identified in this study.

^*b*^ ST, sequence type.

^*c*^ ST newly assigned in this study.

^*d*^ SNPs in comparison to the genome of the first strain (in parentheses).

Some isolates of *A. taiwanensis*, *A. dhakensis*, *A. allosaccharophila*, *E. roggenkampii*, *E. kobei*, *K. michiganensis*, *K. quasipneumoniae* subsp. *similipneumoniae*, and *R. planticola* were initially identified as *A. caviae*, *A. hydrophila*, *A. veronii*, *Enterobacter asburiae*, *Enterobacter bugandensis*, *Klebsiella oxytoca*, *K. pneumoniae*, and *R. ornithinolytica*, respectively, by matrix-assisted laser desorption ionization-time of flight mass spectrometry (MALDI-TOF MS; data not shown).

### *bla*_GES_ carbapenemase genes were predominant among the 75 CPOs

The predominance of *bla*_GESs_ genes (72/75 isolates, 96%) was observed across all 18 bacterial species identified in this study (Table 1). *bla*_GES-5_ was detected in 28 of 75 (37.3%) isolates, and a high frequency (53.6%) of *bla*_GES-5_-positive *Aeromonas* spp. was noted. In addition, a new variant *bla*_GES-48_ (NG_074709.1) and *bla*_GES-24_ were primarily detected in *Aeromonas* spp., corresponding to 10 of 12 (83.3%) *bla*_GES-48_-positive isolates and 8 of 11 (72.7%) *bla*_GES-24_-positive isolates, including two *bla*_GES-24_- and *bla*_IMP-1_-positive isolates. Meanwhile, *bla*_GES-4_, the second most commonly encountered carbapenemase gene (13/75, 17.3%), was harbored by bacterial species belonging to genera other than *Aeromonas*.

In addition to *bla*_GES-48_, five other variants, namely *bla*_GES-47_, *bla*_GES-49_, *bla*_GES-50_, *bla*_GES-51_, and *bla*_GES-54_ (NG_074708.1, NG_076643.1, NG_077975.1, NG_077976.1, and NG_081003.1, respectively), were newly identified in CPOs. The phylogenetic tree constructed from the amino acid sequences of 56 GES variants (Beta-Lactamase Database, available online at http://bldb.eu/BLDB.php?prot=A#GES, accessed on 10 December 2022) classified them broadly into three major clades (Fig. S1). The 32 GES variants mostly sharing a Gly170Ser substitution formed the largest clade, mainly comprising three subclades: one consisting of six GES variants including GES-4, GES-24, and four newly identified variants (GES-47, GES-48, GES-50, and GES-54), one consisting of 15 GES variants including GES-5 and one newly identified variant (GES-51), and one consisting of seven GES variants including GES-6 and one newly identified variant (GES-49). Compared to the GES-4 sequence, GES-47, GES-48, GES-50, and GES-54 harbored Lys104Glu/His116Tyr, Ala21Ser/Lys104Glu, Ala6Thr/Lys104Glu, and Thr109Met mutations, respectively, according to the Ambler numbering scheme (21). GES-51 and GES-49 harbored Ala6Val from GES-5 and GES-6, respectively. Twenty-three new STs were identified among the CPOs, including 19 STs for *Aeromonas* spp. and 2 STs each for *Enterobacter* spp. and *K. quasipneumoniae* subsp. *similipneumoniae*.

As shown in Table 1, the same species belonging to the same STs and harboring the same carbapenemase genes were repeatedly isolated; specifically, *A. caviae* ST1056 carrying *bla*_GES-48_ (8−274 SNP differences), *A. caviae* ST1059 carrying *bla*_GES-5_ (19 and 31 SNP differences), and *K. pneumoniae* subsp. *pneumoniae* ST2791 carrying *bla*_GES-5_ (74−138 SNP differences) were detected in nine, three, and four samples, respectively, across inlets B and C. *bla*_GES-5_-positive *A. caviae* ST1015 (19 and 365 SNP differences), *A. taiwanensis* ST1055 (835 SNP difference), and *A. veronii* bv. *veronii* ST1054 (30 SNP difference) and *bla*_GES-4_-positive *E. cloacae* subsp. cloacae ST1821 (19 SNP difference) were detected in three, two, two, and two samples, respectively, only from inlet B, whereas *bla*_GES-5_-positive *K. quasipneumoniae* subsp. *similipneumoniae* ST1822 (30 and 59 SNP differences), *R. planticola* (115 and 116 SNP differences), and *R. ornithinolytica* (66 SNP difference), *bla*_GES-4_-positive *K. michiganensis* ST95 (13–24 SNP differences) and *R. ornithinolytica* (7–111 SNP differences), and *bla*_IMP-1_-positive *C. freundii* ST116 (12 SNP difference) were detected in three, three, two, four, four, and two samples, respectively, only from inlet C.

### Phylogenetic relationships among 75 CPOs and their AMR-associated gene profiles

**Fig 1:**
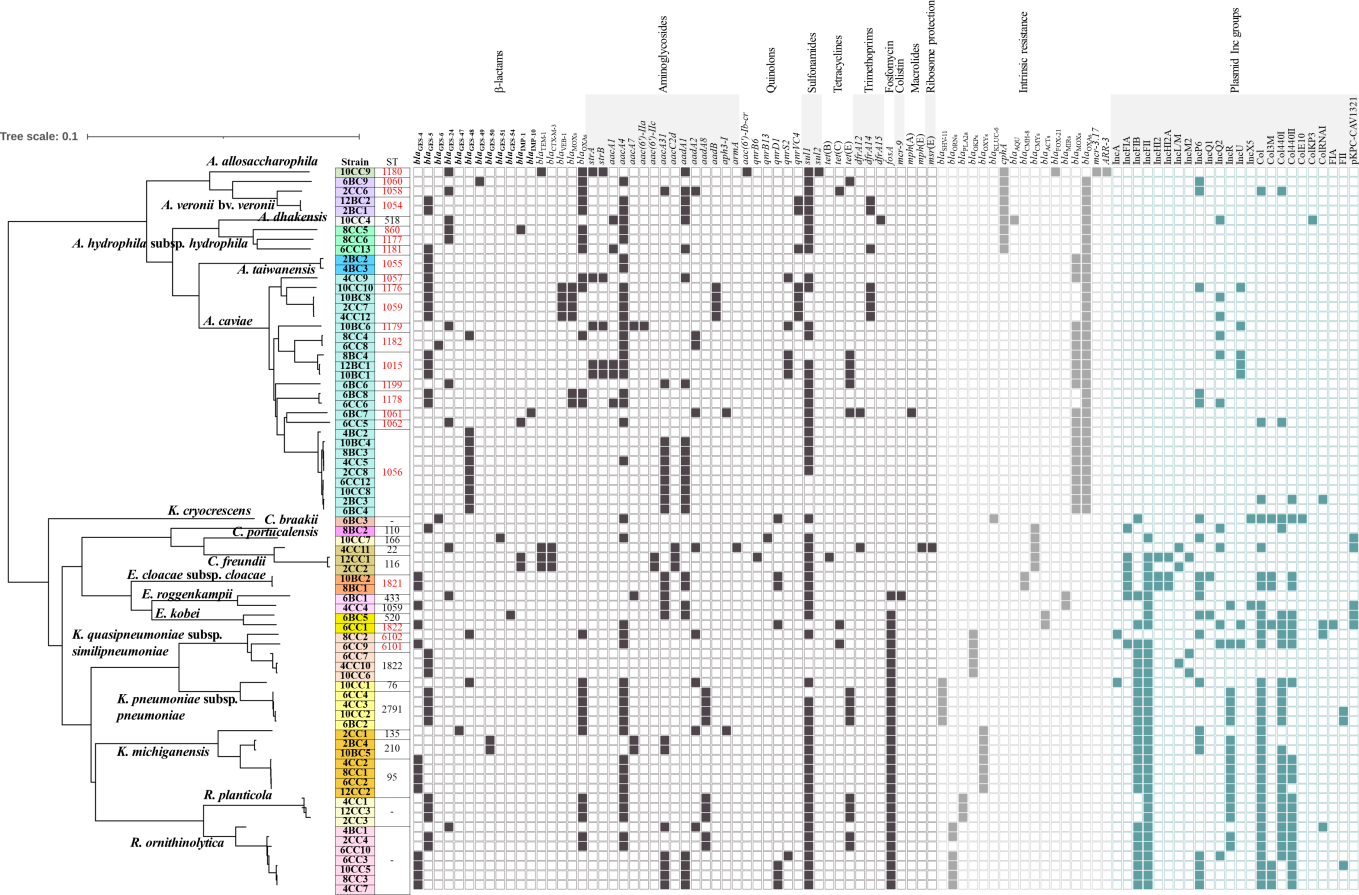
Phylogenetic relationship of 75 CPOs. Core genome neighbour-joining tree (left) was generated by PopPUNK. Strain numbers and their STs are indicated at the right of the tree, with new STs marked in red. The presence (filled squares) or absence (open squares) of antimicrobial resistance-associated genes and plasmid Inc groups among strains is shown.

As presented in Fig. 1, whole-genome clustering via Population Partitioning Using Nucleotide K-mers (PopPUNK) analyses separated the genomes of 75 CPOs into distinct clades that were congruent with their bacterial species and STs. *bla*_CTX-M-3_ was harbored by one ST22 strain (4CC11) and two ST116 strains (12CC1 and 2CC2) of *C. freundii*. Three strains of *A. caviae* ST1059 (10BC8, 2CC7, and 4CC12) and one strain of *A. caviae* ST1176 (10CC10) harbored *bla*_VEB-1_ and *bla*_MOX_, the latter of which was also harbored by two strains of *A. caviae* ST1178 (6BC8 and 6CC6). One *E. roggenkampii* ST433 strain (6BC1) carried *mcr-9. aacA4* and *sul1* were found in 42 (56%) and 57 (76%) isolates, respectively. *fosA* was harbored by 30 of 39 (76.9%) *Enterobactelares* isolates. *cphA* encoding metallo-β-lactamase, which is intrinsic to *Aeromonas* spp., was detected in *A. hydrophila* subsp. *hydrophila*, *A. veronii* bv. *veronii*, *A. dhakensis*, and *A. allosaccharophila*, but it was not detected in *A. caviae* and *A. taiwanensis*.

### Antimicrobial susceptibility of 75 CPOs

The MICs of antimicrobials against 75 CPOs are shown in Table S2. Among the 36 *Aeromonas* stains, 31 GES carbapenemase-producing strains were susceptible to imipenem (MIC ≤ 0.25–4 µg/mL) and meropenem (MIC ≤ 0.12–4 µg/mL). The remaining five strains were resistant to imipenem (*A. hydrophila* subsp. *hydrophila* strains 6CC13 and 8CC6 producing GES-5 and GES-24, respectively) and/or meropenem (*A. hydrophila* subsp. *hydrophila* strain 8CC5 producing GES-24 and IMP-1, 8CC6, *A. caviae* 6CC5 producing GES-24 and IMP-1, and *A. caviae* 6BC7 producing IMP-10). Among the 39 *Enterobacterales* strains, the MICs of imipenem and meropenem varied between ≤0.25 and >8 µg/mL and between ≤0.12 and >8 µg/mL, respectively. Cephalosporin resistance was noted in three strains of *C. freundii*, one of which produced GES-24 and CTX-M-3 extended-spectrum β-lactamase (ESBL) and the other two produced IMP-1 and CTX-M-3 ESBL.

The transformants carrying the new *bla*_GES_ variants, *bla*_GES-47_, *bla*_GES-48_, *bla*_GES-49_, *bla*_GES-50_, *bla*_GES-51_, and *bla*_GES-54_, exhibited slightly higher MICs for imipenem (0.5–2 µg/mL) and meropenem (0.25–1 µg/mL) than NEB10-beta electrocompetent *Escherichia coli* (Table 2). They also had high MICs for ampicillin (>16 µg/mL), piperacillin (MIC 32 to >64 µg/mL), cefazolin (>16 µg/mL), cefotiam (>4 µg/mL), ceftazidime (2 to >16 µg/mL), cefpodoxime (>4 µg/mL), cefmetazole (32 to >32 µg/mL), and flomoxef (8 to >16 µg/mL).

**TABLE 2:** MICs of antimicrobials for carbapenemase-producing organisms harboring newly identified *bla*_GES_ genes, and their transformants^*a*^

Antimicrobial agents	MICs (μg/mL)												
	*bla*_GES-47_		*bla*_GES-48_		*bla*_GES-49_		*bla*_GES-50_		*bla*_GES-51_		*bla*_GES-54_		—
	*K. michiganensis* 2CC1	*E. coli* NEB10-beta	*A. A. caviae* 2CC8	*E. coli* NEB10-beta	*A. A. veronii* bv. *veronii6BC9*	*E. coli* NEB10-beta	*K. michiganensis* 10BC5	*E. coli* NEB10-beta	*C. portucalensis* 10CC7	*E. coli* NEB10-beta	*E. kobei* 6BC5	*E. coli* NEB10-beta	*E. coli* NEB10-beta
Ampicillin	>16	>16	>16	>16	>16	>16	>16	>16	>16	>16	>16	>16	≤2
Piperacillin	>64	>64	16	>64	>64	>64	32	64	64	32	32	32	≤4
SAM	>16–8	>16–8	>16–8	>16–8	>16–8	>16–8	>16–8	>16–8	>16–8	>16–8	>16–8	>16–8	≤2–1
TZP	>64–4	>64–4	16-4	32–4	>64–4	64–4	8-4	8-4	16-4	32–4	16-4	8-4	≤4–4
Cefazolin	>16	>16	>16	>16	>16	>16	>16	>16	>16	>16	>16	>16	1
Cefotiam	>4	>4	>4	>4	>4	>4	4	>4	>4	>4	>4	>4	≤0.5
Ceftazidime	>16	4	8	16	>16	>16	2	2	4	8	16	>16	≤0.5
Cefpodoxime	>4	>4	>4	>4	>4	>4	≤1	>4	>4	>4	4	>4	≤1
Ceftriaxone	4	≤1	16	8	16	8	≤1	≤1	2	2	2	4	≤1
Cefepime	4	≤2	≤2	≤2	≤2	≤2	≤2	≤2	≤2	≤2	≤2	≤2	≤2
Cefmetazole	>32	32	>32	>32	>32	>32	16	32	>32	32	>32	32	≤1
Flomoxef	>16	8	>16	>16	>16	>16	>16	>16	>16	>16	16	16	≤2
Aztreonam	≤2	≤2	≤2	≤2	≤2	≤2	≤2	≤2	≤2	≤2	≤2	≤2	≤2
Imipenem	2	0.5	≤0.25	2	2	0.5	0.5	2	4	2	≤0.25	0.5	≤0.25
Meropenem	4	0.25	0.5	1	1	0.5	0.25	1	4	0.5	0.5	0.5	≤0.12
Gentamicin	≤2	≤2	≤2	4	8	≤2	≤2	≤2	≤2	≤2	≤2	≤2	≤2
Amikacin	≤8	≤8	≤8	≤8	≤8	≤8	≤8	32	≤8	≤8	≤8	≤8	≤8
Minocycline	≤2	≤2	≤2	≤2	4	≤2	≤2	≤2	≤2	≤2	8	≤2	≤2
Levofloxacin	0.5	≤0.12	1	≤0.12	1	≤0.12	0.5	≤0.12	0.5	≤0.12	1	≤0.12	≤0.12
Fosfomycin	≤32	≤32	64	≤32	≤32	≤32	≤32	≤32	≤32	≤32	128	≤32	≤32
SXT	≤9.5–0.5	≤9.5–0.5	19-1	≤9.5–0.5	>38/2	≤9.5–0.5	≤9.5–0.5	≤9.5–0.5	≤9.5–0.5	≤9.5–0.5	19-1	≤9.5–0.5	≤9.5–0.5

^*a*^ MIC, minimum inhibitory concentration; SAM, ampicillin-sulbactam; SXT, sulfamethoxazole-trimethoprim; TZP, piperacillin-tazobactam.

### *bla*_GES-48_-positive *A. caviae* ST1056 occurred at the highest total densities in the influent

**Fig 2:**
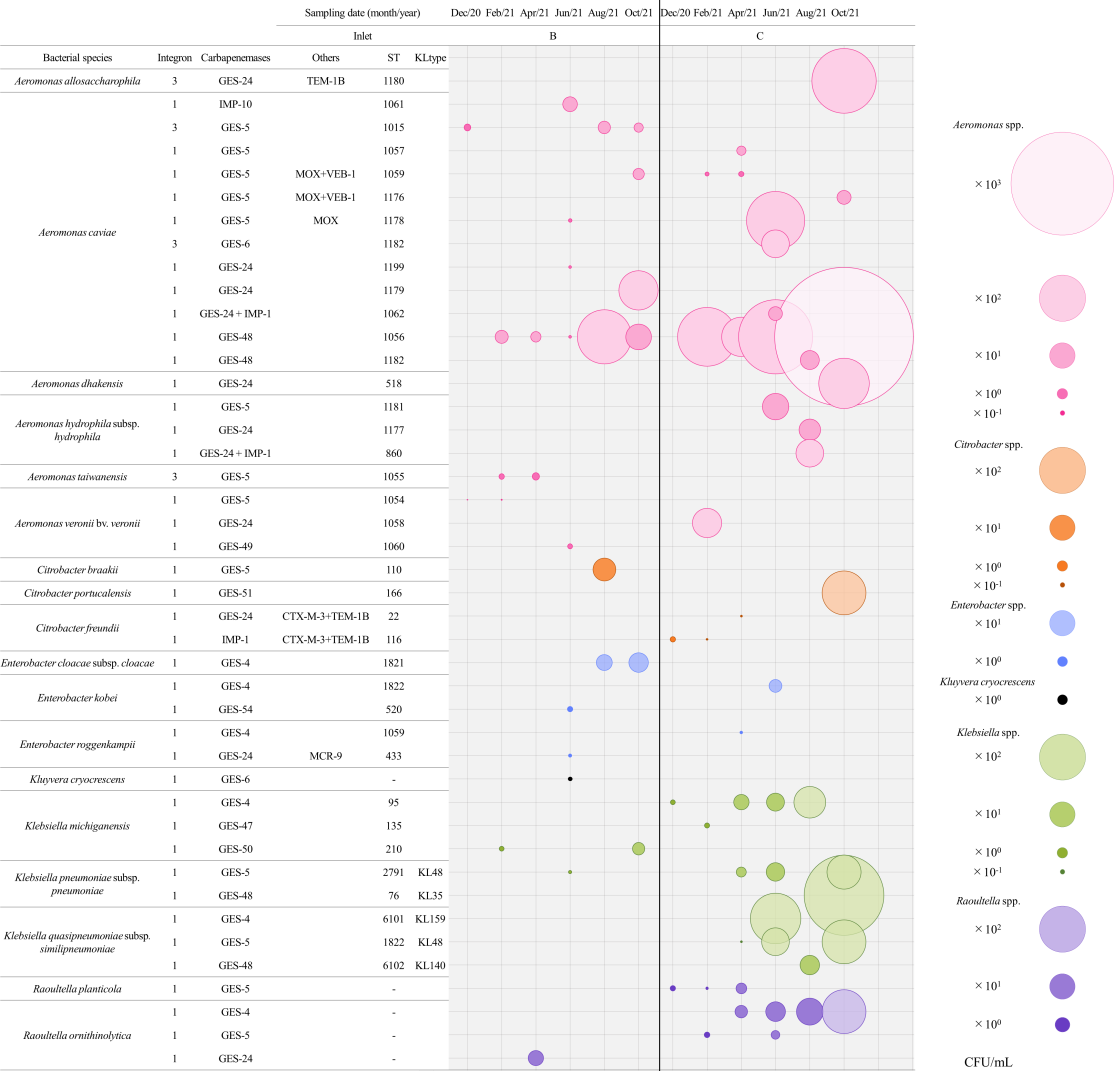
Babble plots showing the abundance (CFU/mL) of CPOs detected in raw influents at inlets B and C. The CFU/mL values were obtained by quantifying organisms of the same bacterial species belonging to the same STs and harboring the same carbapenemase genes.

The abundance (CFU/mL) of CPOs measured bimonthly at inlets B and C is shown in Fig. 2. The bacterial counts of CPOs ranged from 1.0 × 10^−1^ to 3.8 × 10^2^ CFU/mL and from 5.0 × 10^−1^ to 2.5 × 10^3^ CFU/mL for the influent from inlets B and C, respectively, and significant differences in the overall CPO abundance were detected between samples from these two inlets (Wilcoxon signed-rank sum test, *P* = 0.00056). When examining seasonal differences in the abundance of CPOs, no significant differences were detected in samples at each inlet (Friedman test). On a total abundance level, *A. caviae* ST1056 lineage isolates harboring class 1 integron-associated *bla*_GES-48_, which were detected in almost every sampling month at both inlets, represented the most abundant clone (Fig. 2). The occurrence of class 1 integron-associated *bla*_GES-48_ was observed among *A. caviae* ST1182, *K. pneumoniae* subsp. *pneumoniae* ST76, and *K. quasipneumoniae* subsp. *similipneumoniae* ST6102 lineage isolates in the last two sampling months (August and October 2021). Our conjugation experiments between *A. caviae* ST1056 or *A. caviae* ST1182 as donor strains and *K. pneumoniae* subsp. *pneumoniae* ATCC13883 (Rif^r^) or *E. coli* χ1037 (Rif^r^) as the recipient strain failed to obtain any transconjugants harboring *bla*_GES-48_.

**Fig 3:**
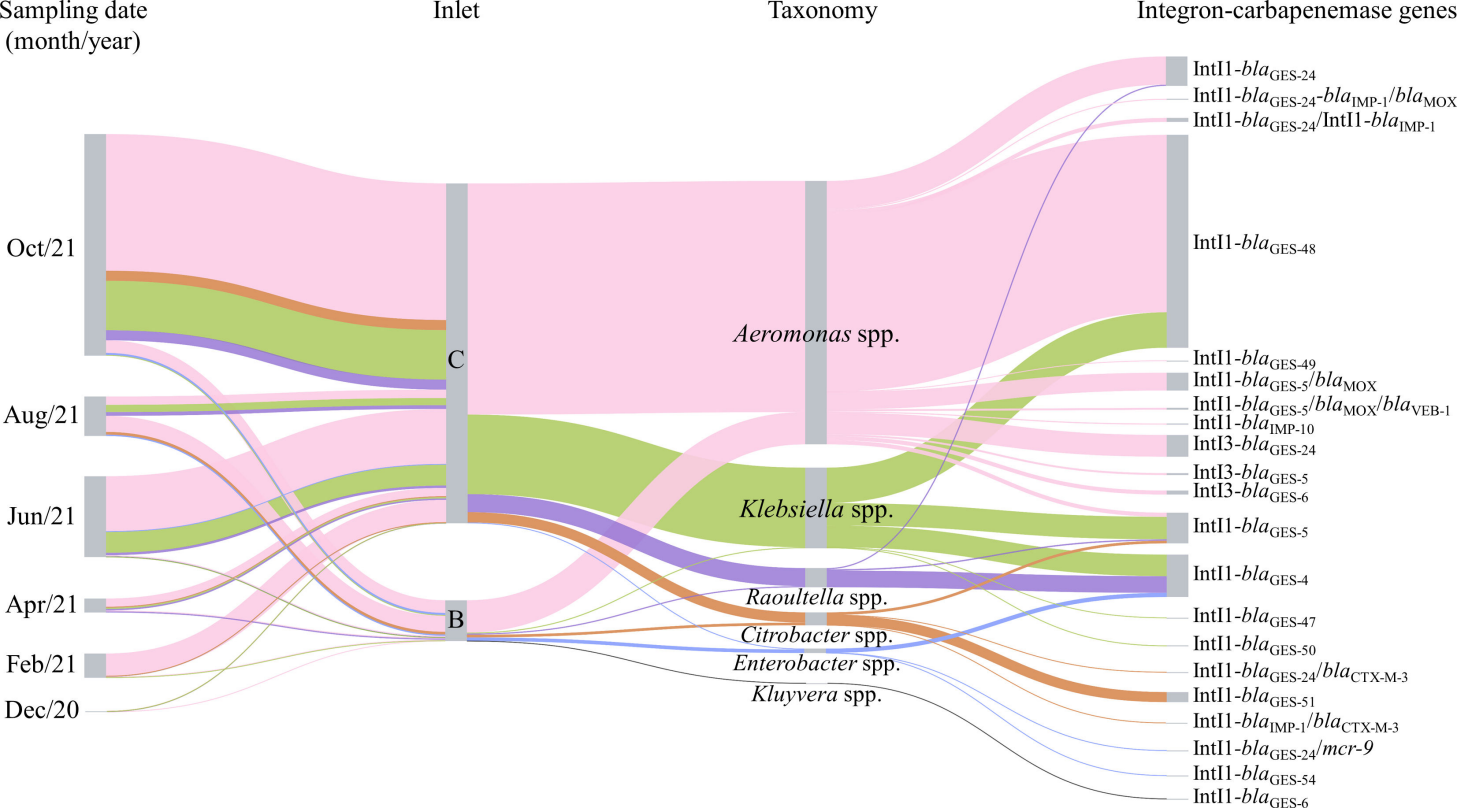
Sankey diagram showing the distribution of the bacterial genera and carbapenemase and integrase genes detected in raw influents across the six sampling months and two inlets. The width of each node is proportional to the abundance (CFU/mL) of the CPOs.

The distribution and linkages among sampling months, wastewater inlets, bacterial genera, and integrons and their associated carbapenemase genes as depicted by the Sankey diagram revealed that *Aeromonas* spp. carrying *bla*_GES-5_, *bla*_GES-6_, *bla*_GES-24_, *bla*_GES-48_, and *bla*_GES-49_ embedded in class 1 or 3 integrons were the most abundant CPOs in the municipal wastewater influent (Fig. 3), followed by *Klebsiella* spp. carrying *bla*_GES-4_, *bla*_GES-5_, *bla*_GES-47_, *bla*_GES-48_, and *bla*_GES-50_ embedded in class 1 integrons.

### Virulence and heavy metal resistance traits of *Aeromonas* spp

Whole-virulome analysis predicted the presence of virulence genes encoding adherence factors [lateral flagella, mannose-sensitive hemagglutinin (Msh) type IV pilus, polar flagella, type IV Tap pili, and type I fimbriae], secretion systems [type II secretion system (T2SS), type III secretion system, and type VI secretion system (T6SS)], and toxins (aerolysin AerA/cytotoxic enterotoxin Act, extracellular hemolysin AHH1, heat-stable cytotonic enterotoxin Ast, and hemolysin HlyA) among different lineage isolates of *Aeromonas* spp. (Fig. S2). The isolates shared Msh type IV pili, polar flagella, type IV Tap pili, T2SS, and hemolysin HlyA genes. Additionally, 6 of the 11 *A*. *caviae* clones (ST1056, ST1061, ST1062, ST1178, ST1179, and ST1199), all *A. veronii* bv. *veronii* clones (ST1054, ST1058, and ST1060), and an *A. taiwanensis* clone (ST1055) had lateral flagellar genes. The T6SS gene was discovered among 4 of the 11 *A*. *caviae* clones (ST1056, ST1061, ST1062, and ST1179), all *A. hydrophila* subsp. *hydrophila* clones (ST860, ST1177, and ST1181), and an *A. dhakensis* clone (ST518). The latter four clones also harbored AerA/Act and AHH1 toxin genes. Heavy metal resistance genes for copper (*copA*), arsenic (*arsB*), and zinc (*zntAB*) were found among *Aeromonas* spp. isolates (Fig. S2). In addition, mercuric resistance genes (*merAEPRT*) were harbored by all three clones of *A. hydrophila* subsp. *hydrophila*, five *A. caviae* clones, one of three clones of *A. veronii* bv. v*eronii*, and one *A. allosaccharophila* clone.

### Genetic context of *bla*_GES_ and *bla*_IMP_ genes

Exploration of the genetic environment surrounding the *bla*_GES_ and *bla*_IMP_ genes in 75 CPOs revealed that these genes were carried in different genetic locations (Table 3). In 23 of 28 *bla*_GES-5_-positive isolates, *bla*_GES-5_ was located in the first gene cassette position immediately downstream of the class 1 integron−integrase gene (*intI1*). Among them, 14 *intI1−bla*_GES-5_-positive isolates harbored class 1 integrons containing the *qacEΔ1−sul1* region in the 3′-conserved segment (3′ CS). The class 1 integron with the structure *intI1−bla*_GES-5_−*bla*_OXA-1042_−*qacL*−3′ CS was shared by two strains each of *A. caviae* ST1178 (6BC8 and 6CC6) and *A. veronii* bv. *veronii* ST1054 (12BC2 and 2BC1), whereas *intI1−bla*_GES-5_−*aacA4−catB10−bla*_OXA-4_−*aadA8*−3′ CS was shared by four *K. pneumoniae* subsp. *pneumoniae* ST2791 strains (4CC3, 6BC2, 6CC4, and 10CC2), three *R. planticola* strains (12CC3, 2CC3, and 4CC1), and two *R. ornithinolytica* strains (2CC4 and 6CC10). Two *E. cloacae* subsp. *cloacae* ST1821 strains (8BC1 and 10BC2) and one *E. roggenkampii* ST1059 strain (4CC4) harbored the class 1 integron containing *intI1–bla*_GES-4_–*aacA31–aadA1–*3′ CS, which was extremely similar to *intI1–bla*_GES-4_–*aacA31–aadA1–aadA1–3*3′ CS contained by four *R. ornithinolytica* strains (4CC7, 6CC3, 8CC3, and 10CC5). The *bla*_GES-48_ gene embedded in a class 1 integron with the structure *intI1–bla*_GES-48_–*aacA31–aadA1–aadA1–3*3′ CS was shared by four *A. caviae* ST1056 strains (2CC8, 4CC5, 8BC3, and 10BC4), whereas one strain (4BC2) from the same lineage carried *intI1–bla*_GES-48_ *–*IS*Pa59–*3′ CS. The genetic context of *bla*_GES-48_ with *intI1–bla*_GES-48_ *–bla*_OXA-129–_ *aadA2–*3′ CS shared by one *K. pneumoniae* subsp. *pneumoniae* ST76 strain (10CC1) and one *K. quasipneumoniae* subsp. *similipneumoniae* ST6102 strain (8CC2) was different from those found in *A. caviae* strains. Of note, the linkage of *bla*_GES-24_, *bla*_GES-6_, and *bla*_GES-5_ with rare class 3 integrons was observed in one *A. allosaccharophila* strain (ST1180), one *A. caviae* strain (ST1182), three *A. caviae* strains (ST1015), and two *A. taiwanensis* strains (ST1055), and the latter six strains carried Tn*402*-like class 3 integrons possessing the complete *tniABQR* transposition module.

**TABLE 3:** Genetic contexts of *bla*_GESs_ and *bla*_IMPs_ in carbapenemase-producing organisms

Bacterial species	Sequence type	Carbapenemase gene	Strain	Genetic context
*A. allosaccharophila*	1,180	*bla*_GES-24_	10CC9	*intI3–bla*_OXA-17_ *–bla*_GES-24_
*A. caviae*	1,061	*bla*_IMP-10_	6BC7	*intI1–bla*_IMP-10_ *–bla*_IMP-10_
	1,015	*bla*_GES-5_	12BC1	*tniABQR–intI3–bla*_GES-5_ *–aacA4*
			8BC4	
			10BC1	*tniABQR–intI3–bla*_GES-5_ *–bla*_GES-5_ *–aacA4*
	1,057	*bla*_GES-5_	4CC9	*intI1–bla*_GES-5_
	1,059	*bla*_GES-5_	2CC7	*intI1–bla*_GES-5_
			4CC12	
			10BC8	
	1,176	*bla*_GES-5_	10CC10	*intI1–bla*_GES-5_ *–bla*_OXA-17_ *–*IS*Pa25–qacEΔ1–sul1*
	1,178	*bla*_GES-5_	6BC8	*intI1–bla*_GES-5_ *–bla*_OXA-1042_ *–qacL–qacEΔ1–sul1*
			6CC6	
	1,182	*bla*_GES-6_	6CC8	*tniABQR–intI3–bla*_GES-6_ *–aadA2–aacA4*
	1,199	*bla*_GES-24_	6BC6	*intI1–bla*_GES-24_ *–aacA31–qacL–qacEΔ1–sul1*
	1,179	*bla*_GES-24_	10BC6	*intI1–aacA7–bla*_GES-24_
	1,062	*bla*_GES-24_/*bla*_IMP-1_	6CC5	*intI1–bla*_GES-24_ *–aacA4–aacA4–bla*_IMP-1_–*sul1*
	1,056	*bla*_GES-48_	2BC3	*intI1–bla*_GES-48_–*aacA31–aadA1–aadA1*
			6BC4	
			10CC8	
			2CC8	*intI1–bla*_GES-48_ *–aacA31–aadA1–aadA1–qacEΔ1–sul1*
			4CC5	
			8BC3	
			10BC4	
			4BC2	*intI1–bla*_GES-48_–IS*Pa59–qacEΔ1–sul1*
			6CC12	*intI1–bla*_GES-48_ *–aacA31–aadA1*
	1,182	*bla*_GES-48_	8CC4	*intI1–bla*_GES-48_
*A. dhakensis*	518	*bla*_GES-24_	10CC4	*intI1–bla*_GES-24_ *–aacA4–catB–aadA1–qacEΔ1–sul1*
*A. hydrophila* subsp. *hydrophila*	1,181	*bla*_GES-5_	6CC13	*intI1–bla*_GES-5_ *–bla*_OXA-1042_
	1,177	*bla*_GES-24_	8CC6	*intI1–bla*_GES-24_ *–bla*_GES-24_
	860	*bla*_GES-24_	8CC5	*intI1–aacA4–catB8–bla*_GES-24_ *–qacEΔ1–sul1*
		*bla*_IMP-1_		*intI1–bla*_IMP-1_ *–bla*_OXA-1053_
*A. taiwanensis*	1,055	*bla*_GES-5_	2BC2	*tniABQR–intI3–bla*_GES-5_ *–aacA4*
			4BC3	*tniABQR–intI3–bla*_GES-5_
*A. veronii* bv. *veronii*	1,054	*bla*_GES-5_	12BC2	*intI1–bla*_GES-5_ *–bla*_OXA-1042_ *–qacL–qacEΔ1–sul1*
			2BC1	
	1,058	*bla*_GES-24_	2CC6	*intI1–bla*_GES-24_ *–aacA31*
	1,060	*bla*_GES-49_	6BC9	*intI1–bla*_GES-49_ *–aacA4–bla*_OXA-129_ *–qacEΔ1–sul1*
*C. braakii*	110	*bla*_GES-5_	8BC2	*intI1–bla*_GES-5_
*C. portucalensis*	166	*bla*_GES-51_	10CC7	*intI1–bla*_GES-51_ *–aacA4–qacEΔ1–sul1*
*C. freundii*	22	*bla*_GES-24_	4CC11	*intI1–bla*_GES-24_
	116	*bla*_IMP-1_	12CC1	*intI1–bla*_IMP-1_ *–aac(6')–IIc–qacEΔ1–sul1*
			2CC2	
*E. cloacae* subsp. *cloacae*	1,821	*bla*_GES-4_	8BC1	*intI1–bla*_GES-4_ *–aacA31–aadA1–qacEΔ1–sul1*
			10BC2	
*E. kobei*	1,822	*bla*_GES-4_	6CC1	*intI1–bla*_GES-4_
	520	*bla*_GES-54_	6BC5	*intI1–bla*_GES-54_ *–aacA31–aadA1–qacEΔ1–sul1*
*E. roggenkampii*	1,059	*bla*_GES-4_	4CC4	*intI1–bla*_GES-4_ *–aacA31–aadA1–qacEΔ1–sul1*
	433	*bla*_GES-24_	6BC1	*intI1–aacA7–bla*_GES-24_ *–catB6–aacA31–qacEΔ1–sul1*
*K. cryocrescens*		*bla*_GES-6_	6BC3	*intI1–bla*_GES-6_ *–aacA4*–ORF*–qacEΔ1–sul1*
*K. michiganensis*	95	*bla*_GES-4_	12CC2	*intI1–bla*_GES-4_ *–aacA4–catB*–ORF*–qacEΔ1–sul1*
			4CC2	
			6CC2	
			8CC1	
	135	*bla*_GES-47_	2CC1	*intI1–bla*_GES-47_
	210	*bla*_GES-50_	2BC4	*intI1–aacA7–bla*_GES-50_ *–catB6–aacA31–qacEΔ1–sul1*
			10BC5	
*K. pneumoniae* subsp. *pneumoniae*	2,791	*bla*_GES-5_	4CC3	*intI1–bla*_GES-5_ *–aacA4–catB10–bla*_OXA-4_ *–aadA8–qacEΔ1–sul1*
			6BC2	
			6CC4	
			10CC2	
	76	*bla*_GES-48_	10CC1	*intI1–bla*_GES-48_ *–bla*_OXA-129_ *–aadA2–qacEΔ1–sul1*
*K. quasipneumoniae* subsp. *similipneumoniae*	6,101	*bla*_GES-4_	6CC9	*intI1–aacA4–*IS*91–intI1–bla*_GES-4_
	1,822	*bla*_GES-5_	4CC10	*intI1–bla*_GES-5_
			6CC7	
			10CC6	
	6,102	*bla*_GES-48_	8CC2	*intI1–bla*_GES-48_ *–bla*_OXA-129_ *–aadA2–qacEΔ1–sul1*
*R. planticola*		*bla*_GES-5_	12CC3	*intI1–bla*_GES-5_ *–aacA4–catB10–bla*_OXA-4_ *–aadA8–qacEΔ1–sul1*
			2CC3	
			4CC1	
*R. ornithinolytica*		*bla*_GES-4_	4CC7	*intI1–bla*_GES-4_ *–aacA31–aadA1–aadA1–qacEΔ1–sul1*
			6CC3	
			8CC3	
			10CC5	
		*bla*_GES-5_	2CC4	*intI1–bla*_GES-5_ *–aacA4–catB10–bla*_OXA-4_ *–aadA8–qacEΔ1–sul1*
			6CC10	
		*bla*_GES-24_	4BC1	*intI1–bla*_GES-24_ *–aadA2–aacA31–qacEΔ1–sul1*

**Fig 4:**
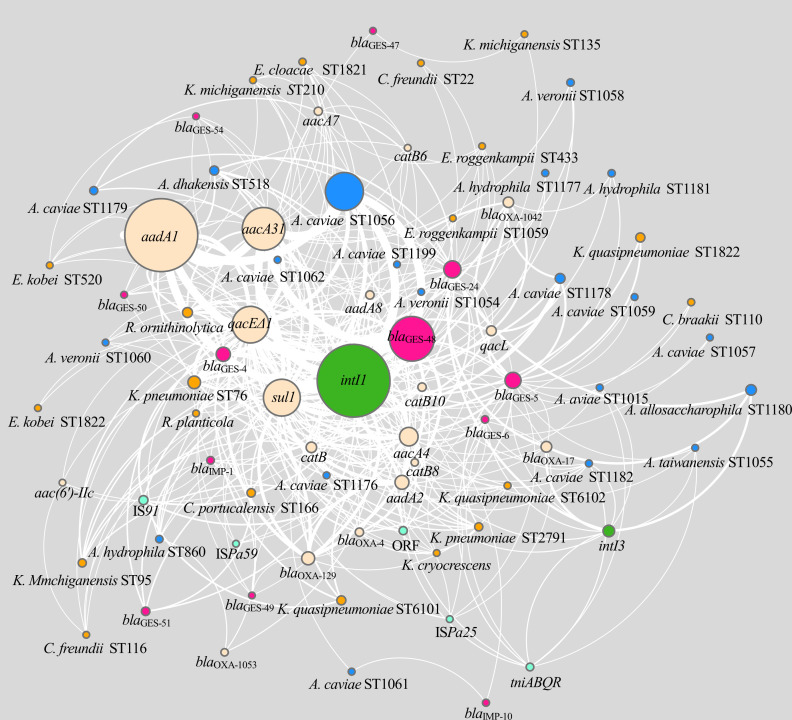
Network analysis depicting co-occurrence relationship among *bla*_GESs_ and *bla*_IMPs_ (pink nodes), class 1 and 3 integron-integrase genes, *intI1* and *intI3* (green nodes), gene cassette composition in these integrons (beige nodes), and species and lineages of *Enterobacterales* (orange nodes) and the genus *Aeromonas* (blue nodes).

Figure 4 shows the network consisting of 78 nodes and 777 edges depicting the co-occurrence patterns among *bla*_GES_ and *bla*_IMP_ gene cassettes in class 1 and 3 integrons and bacterial lineages. The topological analysis revealed that the top 15 ranking genes (*intI1*, *sul1*, *qacEΔ1*, *aacA4*, *bla*_GES-5_, *aacA31*, *aadA1*, *bla*_GES-24_, *bla*_GES-48_, *bla*_GES-4_, *aadA8*, *bla*_OXA-4_, *catB10*, *intI3*, and *aadA2*) with node degrees exceeding the average value and higher betweenness centrality and closeness centrality values (greater than the median of the nodes in the network) were considered hub nodes. Clustering analysis by MCODE identified three important cluster subnetworks of highly intraconnected nodes. The first cluster, consisting of 77 nodes and 774 edges with an MCODE score of 9.692, mainly depicted densely connected nodes of class 1 integron-associated genes (*intI1*, *qacEΔ1*, and *sul1*), carbapenemase genes, other resistance genes, and diverse host species and lineages. The second cluster (32 nodes and 99 edges with an MCODE score of 3.879) featured a significant relationship among carbapenemase genes (*bla*_GES-24_, *bla*_GES-48_, *bla*_GES-54_, and *bla*_IMP-1_), several other resistance genes, and mainly *Aeromonas* and *Enterobacter* spp. as host species. The third cluster (11 nodes and 27 edges with an MCODE score of 3.333) mainly featured a relationship among *bla*_GES-48_, *aadA2*, and *Klebsiella* spp.

## DISCUSSION

This study revealed the occurrence and persistence of CPOs comprising a diverse range of bacterial species and lineages in wastewater influent from inlets B and C connected to combined sewer systems collecting both primarily domestic/industrial wastewater and urban runoff rainwater. CPOs carrying *bla*_GES_ carbapenemase genes were overwhelmingly dominant, accounting for 72 of 75 isolates (96%). Our finding is congruent with previous reports documenting the predominance of *bla*_GES_ genes among CPOs in raw wastewater samples from WWTPs (15, 22). However, our results are unique in that various *bla*_GES_ variants, including six newly identified variants, were associated with CPOs, and *Aeromonas* spp. were the most dominant CPO species. In addition, CPOs were not detected in any raw influent from inlet A connected to the separated sewer systems carrying only domestic/industrial wastewater. Thus, the load of water washing the ground surface, including rainwater, contaminated with CPOs carrying *bla*_GES_ carbapenemase genes from unrecognized environmental sources could contribute significantly to their detection in the wastewater influent from inlets B and C. In particular, in recent years, the volume of runoff has often exceeded the capacity of the combined sewer system due to localized heavy rainfall resulting from abnormal weather conditions. This can lead to the backflow of wastewater, resulting in the spread of CPOs throughout the entire system. However, one limitation of the study is the lack of data on this type of water monitoring. The *bla*_GES_ carbapenemase genes (*bla*_GES-5_, *bla*_GES-6_, *bla*_GES-16_, and *bla*_GES-24_) carried by diverse *Enterobacterales* and non-*Enterobacterales* species have been documented in aquatic environments, such as urban streams (23), river water (24, 25), coastal water (26, 27), urban ponds (28), lake water (29), wastewater (30), and hospital sewage (19, 31, 32). In this study, we could not identify potential sources of the *bla*_GES_ carbapenemase genes. However, the possible presence of these CPOs of environmental origin is also supported by the findings that *Aeromonas* spp., which are ubiquitous in aquatic environments and soils and capable of rapidly colonizing various niches and hosts (33), permitting them to serve as potential environmental reservoir bacteria for carbapenemase genes, were the most predominant CPO species harboring *bla*_GES_ carbapenemase genes. *bla*_GES_ genes remain rare in clinical isolates, accounting for 2.1% (5/240 isolates) among isolates positive for carbapenemase genes according to the carbapenem-resistant *Enterobacteriaceae* surveillance in the Infectious Agents Surveillance System, 2020, in Japan (available online at https://www.niid.go.jp/niid/ja/cre-m/cre-iasrd/11520-511d01.html). Thus, the normal human microbiota is unlikely to be a source of the *bla*_GES_ carbapenemase genes primarily detected in wastewater influent in our study.

In this study, we described the occurrence of multiple *bla*_GES_ variants, with *bla*_GES-5_ being the most prevalent in 36 *Aeromonas* spp. strains mostly belonging to 19 newly identified STs and in 39 *Enterobacterales* strains including *Enterobacter*, *Klebsiella*, and *Raoultella* spp. Furthermore, six variants of *bla*_GES_ carbapenemase genes, namely *bla*_GES-47_, *bla*_GES-48_, *bla*_GES-49_, *bla*_GES-50_, *bla*_GES-51_, and *bla*_GES-54_, were newly identified. Compared to the sequence of GES-1, GES-49 and GES-54 shared the amino acid substitution Glu104Lys; GES-47, GES-48, GES-50, and GES-54 shared the amino acid substitution Met62Thr; and an amino acid change at position 6 was shared by GES-49 and GES-51 (Ala6Val) and GES-50 (Ala6Thr), in addition to the Gly170Ser substitution identified in all six *bla*_GES_ variants. The Gly170Ser substitution within the Ω-loop region displays carbapenem-hydrolyzing activity (34). This substitution has also been associated with increased catalytic efficiency against cephamycin (cefoxitin), whereas Glu104Lys was linked to enhanced hydrolytic activity toward oxyimino-cephalosporins (35). The Met62Thr substitution does not significantly affect the MICs of β-lactams for *E. coli* clones (36). The Ala6Thr substitution in the leader peptide was associated with higher MICs for β-lactams (cefotaxime, aztreonam, and imipenem) (37). The phenotypic effects of these resistance-related substitutions did not vary substantially, as all *E. coli* transformants gaining these new *bla*_GES_ variants showed susceptibility or intermediate resistance to imipenem and meropenem with MIC ranges of 0.5–2 and 0.25–1 µg/mL, respectively.

Class 1 integrons, being capable of capturing AMR genes, play a particularly important role in the acquisition and dissemination of multidrug resistance among Gram-negative bacteria (38). *bla*_GES_ and *bla*_IMP_ genes are frequently associated with class 1 integrons in *Enterobacterales*, *Pseudomonas*, and *Aeromonas* spp. of clinical and aquatic environmental origin (17, 20, 30, 39). These carbapenemase genes have been less commonly associated with class 3 integrons, but our recent study revealed the linkage of *bla*_GES-24_, which was predominant among CPO isolates in hospital wastewater, with rare Tn*402*-like class 3 integrons (19). In the present study, *bla*_GES_ and/or *bla*_IMP_ genes carried by 29 *Aeromonas* spp. and 39 *Enterobacterales* strains were embedded in class 1 integrons, whereas *bla*_GES_ genes (*bla*_GES-5_, *bla*_GES-6_, and *bla*_GES-24_) carried by the remaining seven *Aeromonas* spp. strains were mostly embedded in Tn*402*-like class 3 integrons. Although structural diversity of gene cassette arrays was noted in the class 1 integrons carried by these 68 CPOs, several shared gene cassettes were persistent in wastewater environment, including one between *A. veronii* bv. *veronii* ST1054 and *A. caviae* ST1178 strains, one among *K. pneumoniae* subsp. *pneumoniae* ST2791, *R. planticola*, and *R. ornithinolytica*, and one between *E. cloacae* subsp. *cloacae* ST1821 and *E. roggenkampii* ST1059 (*R. ornithinolytica* also carried an extremely similar one). *A. caviae* ST1015 and A. *taiwanensis* ST1055 strains shared the class 3 integron *tniABQR–intI3–bla*_GES-5_–*aacA4*. Thus, these integrons, by association with transposons and plasmids, might contribute to the dissemination of *bla*_GES_ carbapenemase genes through horizontal gene transfer events (40) within different species of *Aeromonas* or *Enterobacter*/*Klebsiella*/*Raoultella*.

Quantitative analysis of CPOs revealed that the repeated detection of *bla*_GES-48_-positive *A. caviae* ST1056 from both inlets B and C ranked the total number of this bacterial clone highest in the wastewater influent, suggesting the constant presence of this clone in the combined sewer wastewater. Biofilm formation mediated by polar flagella, lateral flagella, T6SS, etc. might also allow this clone to become a resident organism in the sewer system. However, there might be a missing link to explain the occurrence of the *bla*_GES-48_-positive *A. caviae* ST1056 strains and the *bla*_GES-5_-positive *A. caviae* ST1059, *A. caviae* ST1178, and *K. pneumoniae* subsp. *pneumoniae* ST2791 strains in influent from both inlets B and C. Interestingly, eight of nine *A. caviae* ST1056 strains harbored identical or extremely similar class 1 integron cassettes around the *bla*_GES-48_ genes (*intI1–bla*_GES-48_–*aacA31–aadA1–aadA1–qacEΔ1–sul1*), which were different from the structure of class 1 integron with *bla*_GES-48_ carried by the remaining *A. caviae* ST1056 strain (*intI1–bla*_GES-48_–IS*Pa59–qacEΔ1–sul1*) and by one each of *K. pneumoniae* subsp. *pneumoniae* ST76 and *K. quasipneumoniae* subsp. *similipneumoniae* ST6102 strains (*intI1–bla*_GES-48_–*bla*_OXA-129_–*aadA2–qacEΔ1–sul1*). *bla*_GES-48_ could not be transferred via conjugation between *A. caviae* ST1056 and *K. pneumoniae* subsp. *pneumoniae* ATCC13883 or *E. coli* χ1037; thus, *bla*_GES-48_ harbored by *A. caviae* ST1056 is likely associated with an *Aeromonas*-specific plasmid.

In this study, a representative colony was selected from a minimum of three colonies with the same colony morphology, the same bacterial species, and the same carbapenemase genes. This selection process of representative colonies can result in the successful detection of different ST types within the same CPO species sharing the same carbapenemase genes and the successful tracking of the presence of CPOs belonging to multiple STs in wastewater influents. However, these representative colonies are not guaranteed to be an exact match to the corresponding bacterial populations in terms of bacterial species and genetic types, which may limit the quantitative detection of CPOs under the conditions studied.

In summary, our study revealed the high prevalence and persistence of *bla*_GES_ carbapenemase genes among CPOs isolated from influent inlets connected to combined sewer systems. *Aeromonas* spp. carrying *bla*_GES-5_, *bla*_GES-6_, *bla*_GES-24_, *bla*_GES-48_, and *bla*_GES-49_ embedded in class 1 or 3 integrons stood out as the most abundant CPOs, followed by *Klebsiella* spp. carrying *bla*_GES-4_, *bla*_GES-5_, *bla*_GES-47_, *bla*_GES-48_, and *bla*_GES-50_ embedded in class 1 integrons. The presence of diverse integrons, *bla*_GES_ variants, and host clones of *Aeromonas* spp., *Enterobacter* spp., *Klebsiella* spp., and *Raoultella* spp. represents the plasticity of these genetic elements, which might allow integrons to capture and disseminate such *bla*_GES_ variants and facilitate the adaptability of CPOs to the environment. This study shed light on the great potential of the environment in holding *bla*_GES_ carbapenemase genes and promoting their genetic variability.

## MATERIALS AND METHODS

### Sample collection and bacterial isolation

One sample each of raw influent from three different inlets, namely, inlet A receiving separated sewer lines and inlets B and C receiving combined sewer lines, and treated effluent from the WWTP outlet were collected once every 2 months on rain-free days from December 2020 to October 2021 from the municipal WWTP located in Matsumoto City, Nagano Prefecture, Japan (Fig. S3). The WWTP processes approximately 82,200 m^3^ of wastewater per day from 124,700 inhabitants, comprising 43,980, 24,540, and 56,180 inhabitants for inlets A, B, and C, respectively. Simultaneously, river water samples from sites 1.35-km upstream (36°13′54.7″N, 137°57′11.0″E) and 0.56-km downstream (36°14′56.0″N, 137°57′4.7″E) from the effluent outlet were collected (Table S1). Each sample (approximately 1,000 mL) was placed in cleaned polypropylene bottles and immediately transported in a cooler box with sufficient ice packs to our laboratory for processing within 2 h after obtaining the samples. A volume of 250 mL from the influent was concentrated 100-fold via centrifugation at 3,500 rpm for 20 min, after which the pellet was suspended in 2.5 mL of sterile phosphate-buffered saline (PBS, pH 7.4). For effluent and river water samples, 600 mL were filtered through a 0.45 µM membrane (HAWP04700; Millipore). Then, the filter was cut into small pieces and placed in 6 mL of sterile PBS to prepare a bacterial cell suspension. Finally, a 1,000× concentrated suspension was obtained via centrifugation at 15,000 rpm for 10 min.

The 100× concentrated influent samples were serially diluted using sterile PBS, and 100 µL of each dilution was spread onto MacConkey agar (Eiken Chemical Co., Tokyo, Japan) containing 1 µg/mL imipenem (Sigma-Aldrich Japan, Tokyo, Japan) and CHROMagar mSuperCARBA (Kanto Chemical, Tokyo, Japan), followed by incubation at 37°C overnight. In the same manner, 100 µL aliquots of 1,000× concentrated suspensions obtained from effluent and river water samples were spread directly on the same agar plates.

For each sample, bacterial colonies exhibiting similar morphological features were counted using only agar plates yielding approximately 300 visible colonies, enabling us to calculate the number of CFU per milliliter. Then, a minimum of three well-isolated colonies from the bacterial population with similar colony morphology were individually subcultured onto Mueller−Hinton agar (Eiken) and subjected to MALDI-TOF MS with the Bruker BioTyper database and software version 3.1 (Bruker Daltonics Japan, Yokohama, Japan) using a cutoff of ≥2.00 for species-level identification. Intrinsically carbapenem-resistant bacterial species, including *Morganella morganii*, *Providencia rettgeri*, and *Stenotrophomonas maltophilia*, were excluded from further analysis after PCR confirmation of the absence of acquired carbapenemase genes.

### Detection of carbapenemase genes

The carbapenemase genes *bla*_IMP_ *, bla*_NDM_ *, bla*_KPC_ *, bla*_GES_, and *bla*_OXA-48_ were screened by PCR and identified by DNA sequencing (19). After confirming by MALDI-TOF MS that at least three colonies representing a population of bacteria with similar colony morphology were the same bacterial species and harbored the same carbapenemase genes, a representative strain was selected from these colonies. Plural representative isolates identified as *K. pneumoniae* sharing the same carbapenemase genes obtained from the same agar plate were further differentiated by analyzing *rpoB* sequences (41).

### Antimicrobial susceptibility testing

The MICs of the carbapenemase-producing isolates were determined by the broth microdilution method recommended by the Clinical and Laboratory Standards Institute (CLSI) using Dry Plate DP41 (Eiken), and the results were interpreted using CLSI document M100-ED32 (42). The MICs of faropenem (Sigma-Aldrich), ertapenem (Fujifilm Wako Pure Chemical Co., Osaka, Japan), colistin (Fujifilm), and tigecycline (Tokyo Chemical Industry Co., Tokyo, Japan) were determined using in-house prepared panels according to the CLSI broth microdilution method. *E. coli* ATCC25922 was used as a quality control strain.

### Transformation experiments

Transformation of NEB 10-beta electrocompetent *E. coli* (DH10B derivative, New England Biolabs, Tokyo, Japan) with plasmid DNA extracted from each of the carbapenemase-producing isolates harboring new variants of the *bla*_GES_ genes (*bla*_GES-47_, *bla*_GES-48_, *bla*_GES-49_, *bla*_GES-50_, *bla*_GES-51_, and *bla*_GES-54_) was performed by electroporation. Transformants were selected on LB agar plates containing ampicillin (50 µg/mL), and the presence of *bla*_GES_ genes and the absence of other β-lactamase genes were confirmed by PCR and sequencing. The MICs for the transformants were determined in the same manner described above.

### Transferability of *bla*_GES-48_ from *A. caviae* to *Enterobacterales*

The broth mating assay was used to investigate the transferability of *bla*_GES-48_ from the parental isolates, *A. caviae* ST1056 strain 2BC3 and *A. caviae* ST1182 strain 8CC4 to *K. pneumoniae* subsp. *pneumoniae* ATCC13883 (Rif^r^) or *E. coli* χ1037 (Rif^r^). Conjugation was performed at 25°C and 37°C, and transconjugants were selected on rifampicin-containing (100 µg/mL) LB agar plates supplemented with faropenem (16 µg/mL) and ampicillin (50 µg/mL) for the former and latter recipients, respectively.

### WGS and bioinformatics

Genomic DNA was extracted using lysozyme and proteinase K and purified using an Agencourt AMpure XP kit (Beckman-Coulter Life Sciences, Brea, CA, USA) according to the manufacturer’s instructions. To construct DNA libraries, 5× whole-genome sequencing (WGS) Fragmentation Mix and 5× WGS Ligase Mix (Enzymatics, Beverly, MA, USA) were used. Pooled libraries were subjected to 350- to 800-bp size selection using the BluePippin system (Sage Science, Inc., Beverly, MA, USA). The pooled libraries were sequenced on the Illumina HiSeq X Five platform (Illumina Inc., San Diego, CA, USA) using the 150-bp paired-end method. A small portion of the CPOs were subjected to WGS by the 150-bp paired-end method using the NovaSeq6000, MiSeq, and MiniSeq platforms (Illumina). The de novo assembly of short reads was conducted using Shovill v1.1.0 (https://github.com/tseemann/shovill).

Pairwise ANIm was calculated using JspeciesWS (http://jspecies.ribohost.com/jspeciesws/), and 95%–96% ANI was used as the threshold for defining species. *A. allosaccharophila* CECT 4199 (GCA_000819685.1), *A. caviae* NCTC 12244 (GCA_900476005.1), *A. dhakensis* F2S2-1 (GCA_001673685.1), *A. hydrophila* subsp. *hydrophila* ATCC 7966 (GCA_000014805.1), *A. taiwanensis* LMG 24683 (GCA_000820165.1), *A. veronii* bv. *veronii* CECT 4257 (GCA_000820225.1), *C. braakii* ATCC 51113 (GCA_002075345.1), *C. freundii* ATCC 8090 (GCA_011064845.1), *C. portucalensis* A60 (GCA_002042885.1), *E. cloacae* subsp. *cloacae* ATCC 13047 (GCA_000025565.1), *E. kobei* DSM 13645 (GCA_001729765.1), *E. roggenkampii* DSM 16690 (GCA_001729805.1), *K. cryocrescens* NBRC 102467 (GCA_001571285.1), *K. michiganensis* 10–5242 (GCA_000247835.1), *K. pneumoniae* subsp. *pneumoniae* ATCC 13883 (GCA_000788015.1), *K. quasipneumoniae* subsp. *similipneumoniae* ATCC 700603 (GCA_003181175.1), *R. ornithinolytica* ATCC 31898 (GCA_001598295.1), and *R. planticola* ATCC 33531 (GCA_000735435.1) served as the reference genomes were used for ANIm analyses. Assembled genomes were scanned against ResFinder 4.1, PlasmidFinder 2.1, and CSI Phylogeny 1.4 available from the Center for Genomic Epidemiology (http://www.genomicepidemiology.org). Multilocus sequence typing (MLST) was performed using mlst v2.22.0 (https://github.com/tseemann/mlst). The capsule and outer lipopolysaccharide loci of *K. pneumoniae* species complex isolates were typed using Kaptive v2.0.0 (https://github.com/katholt/kaptive). In *Aeromonas* spp., virulence-associated genes were analyzed using VFanalyzer from the virulence factors database (http://www.mgc.ac.cn/VFs/), and heavy metal resistance genes were explored manually using WGS data.

Genome annotation was achieved using Prokka 1.14.6 (https://github.com/tseemann/prokka). Whole-genome clustering based on k-mer mash distances among genomes was conducted using PopPUNK (43) on a Galaxy ARIES-based platform (Galaxy Version 1.1). iTOL v6 (https://itol.embl.de/) was used to annotate and visualize the tree. The genetic context of the carbapenemase gene was investigated by PCR mapping and sequencing. The co-occurrence relationships among integrons, resistance genes, and bacterial strains were visualized in a network using Cytoscape version 3.9.1 (44). The Cytoscape plugin cytoHubba (v0.1) (45) and MCODE (v2.0.2) (46) were used to predict hub nodes based on topological parameters and the identification of highly intraconnected clusters in a network, respectively. New STs were assigned by the Institut Pasteur MLST database or PubMLST database. New allele numbers for *bla*_GES_ carbapenemase genes were assigned by NCBI.

### Statistical analysis

The abundance of CPOs was not normally distributed according to the D’Agostino−Pearson omnibus normality test; thus, the occurrence of CPOs in influent samples from inlets B and C was compared using the nonparametric Wilcoxon signed-rank sum test. The Friedman test was used to investigate differences in the occurrence of CPOs between sampling months. *P* ≤ 0.05 indicated statistical significance.

## Data Availability

All raw and assembled sequence data for 75 CPOs have been deposited in the DDBJ/EMBL/GenBank database under the umbrella project PRJDB14710 that encompasses two primary submission projects, PRJDB14712 and PRJDB13738. The complete nucleotide sequences of *bla*_GES-47_, *bla*_GES-48_, *bla*_GES-49_, *bla*_GES-50_, *bla*_GES-51_, and *bla*_GES-54_ have been deposited in the DDBJ/EMBL/GenBank nucleotide sequence database under accession numbers NG_074708.1, NG_074709.1, NG_076643.1, NG_077975.1, NG_077976.1, and NG_081003.1, respectively.

## SUPPLEMENTAL MATERIAL

Figs. S1 to S3, Tables S1 to S2 spectrum.02188-23-s0001.pdf Supplementary data.

ASM does not own the copyrights to Supplemental Material that may be linked to, or accessed through, an article. The authors have granted ASM a non-exclusive, world-wide license to publish the Supplemental Material files. Please contact the corresponding author directly for reuse.
