# Supplementary material for: Municipal wastewater monitoring revealed the predominance of bla GES genes with diverse variants among carbapenemase-producing organisms: high occurrence and persistence of Aeromonas caviae harboring the new bla GES variant bla GES-48
Source: Microbiol Spectr. 2023 Oct 9;11(6):e02188-23. doi: 10.1128/spectrum.02188-23 (PMC10715227; doi:10.1128/spectrum.02188-23)
Supplement: Figs. S1 to S3, Tables S1 to S2 — Supplementary data. [file spectrum.02188-23-s0001.pdf]

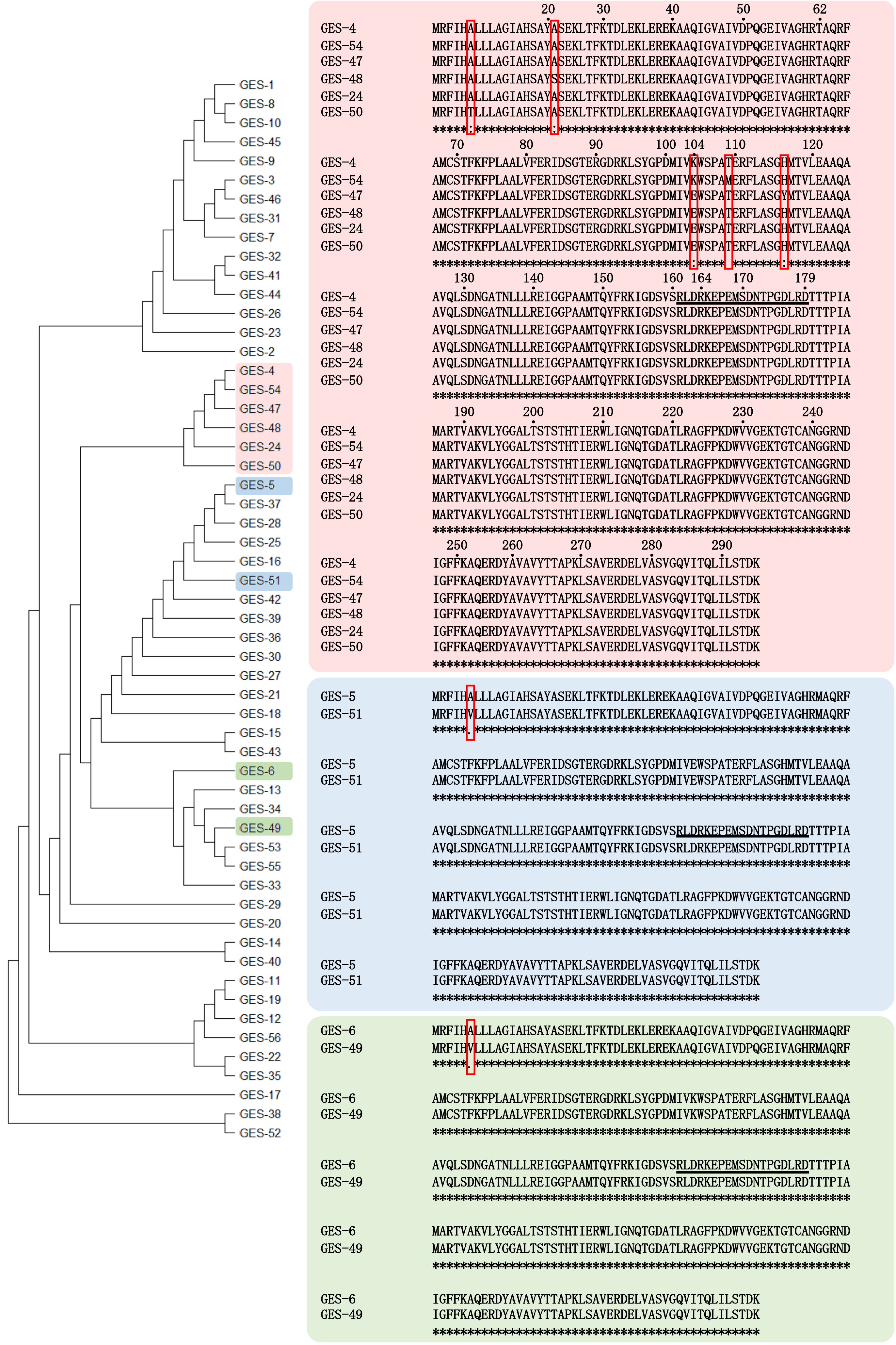

Fig. S1. Comparison of the amino acid sequence of GES-47, -48, -50, and -54, GES-51, and GES-49 to those of GES-4, -5, and -6, respectively. The amino acids of the omega loop are underlined.

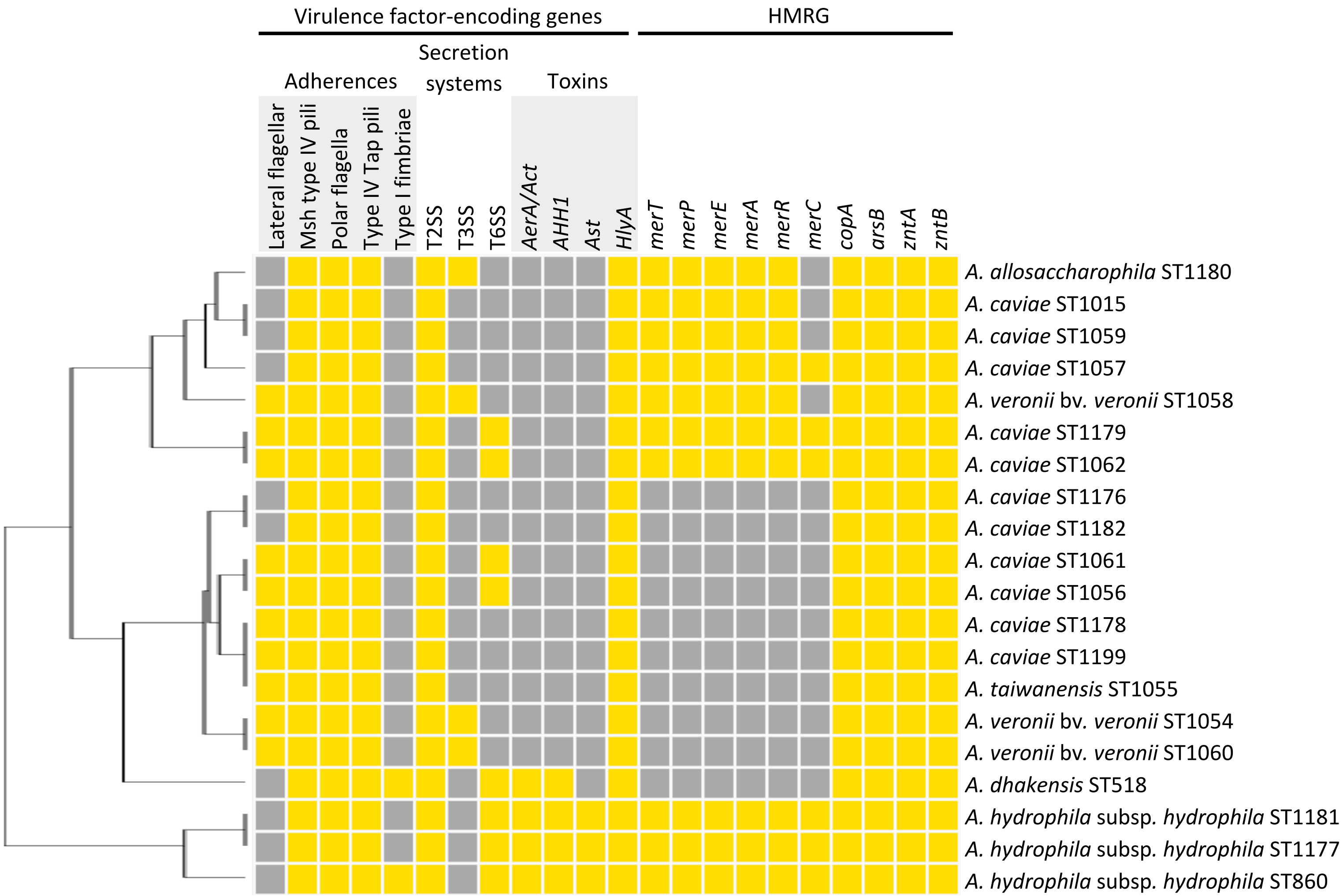

Fig. S2. Profiles of virulence factor-encoding genes and heavy metal resistance genes (HMRG) among 20 different lineage isolates of *Aeromonas* spp. Heat map, representing the presence (yellow) or absence (gray) of genes was generated using Morpheus software (https://software.broadinstitute.org/morpheus/).

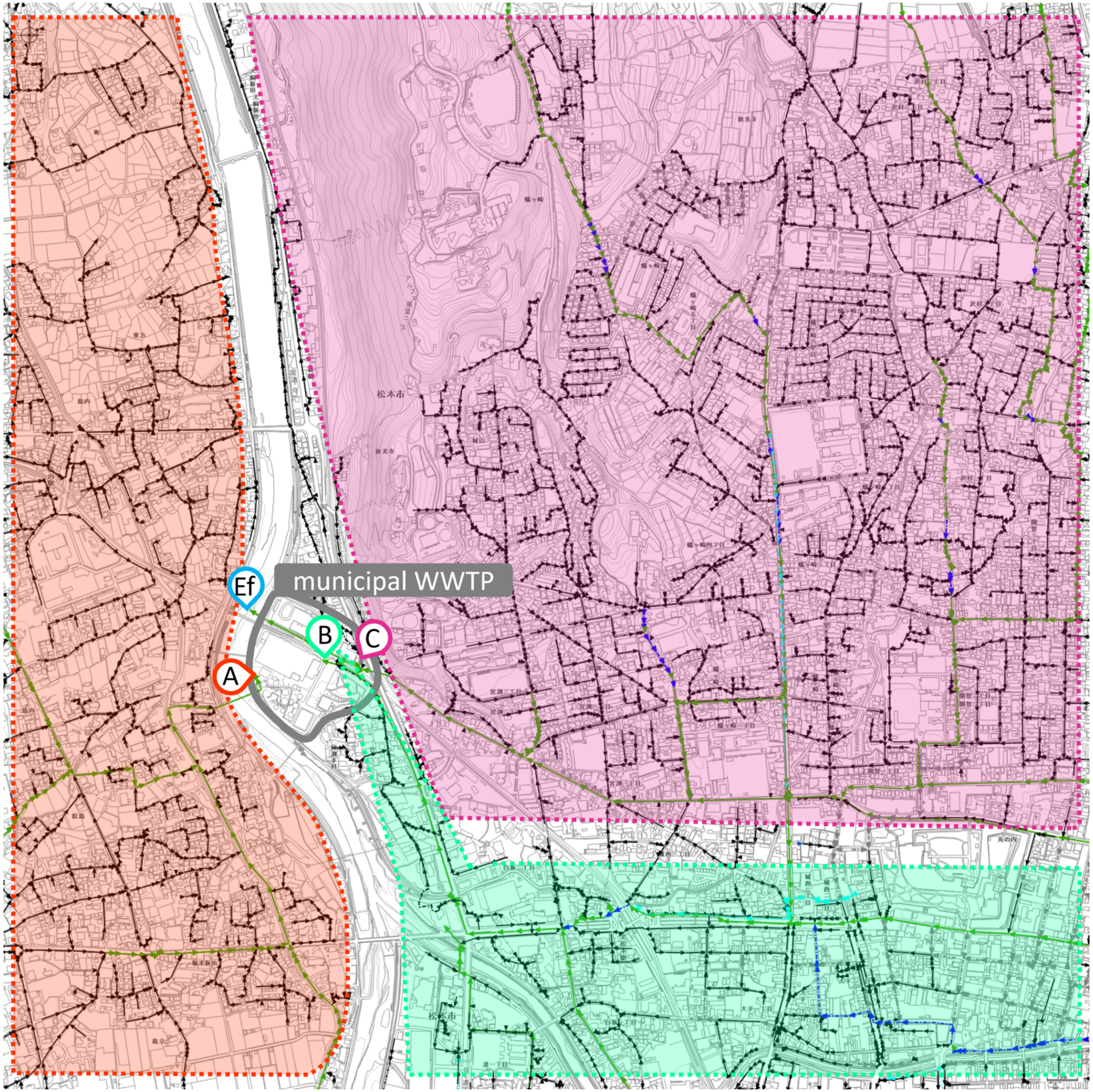

Fig. S3. Sampling points at the municipal wastewater treatment plant located in Matsumoto City, Nagano Prefecture, Japan.
A, inlet A receiving separated sewer line in the western area of the city (orange); B, inlet B receiving combined sewer line in the southeastern area of the city (green); C, inlet C receiving combined sewer line in the northeastern area of the city (pink); Ef, effluent outlet.

TABLE S1. Measurement results of temperature and pH at sampling points

Sample	Sampling point (latitude/longitude)	Parameter	Sampling date (month/year)						Average ± SD
			Dec-20	Feb-21	Apr-21	Jun-21	Aug-21	Oct-21	
Influent A	Inlet A (36°14'38.0"N 137°57'02.9"E)	Temperature	16℃	14℃	16℃	20℃	23℃	22℃	18.5 ± 3.4
		pH	7.53	7.55	7.51	7.31	7.23	7.29	7.4 ± 0.1
Influent B	Inlet B (36°24'30.3"N 137°95'21.0"E)	Temperature	17℃	13℃	17℃	20℃	22℃	21℃	18.3 ± 3.0
		pH	7.47	7.41	7.29	7.36	7.37	7.06	7.3 ± 0.1
Influent C	Inlet C (36°14'35.2"N 137°57'12.0"E)	Temperature	18℃	13℃	18℃	21℃	24℃	23℃	19.5 ± 3.7
		pH	7.53	7.29	7.28	7.09	7.19	7.17	7.3 ± 0.1
Effluent	36°14'38.0"N 137°57'02.9"E	Temperature	17℃	14℃	18℃	21℃	25℃	22℃	19.5 ± 3.6
		pH	7.22	6.84	6.95	6.71	7.01	7.06	7.0 ± 0.2
River water (1.35 km upstream from the effluent outlet)	36°13'54.7"N 137°57'11.0"E	Temperature	11℃	9℃	10℃	17℃	18℃	15℃	13.3 ± 3.5
		pH	7.6	7.4	8.1	5.9	7	6	7.0 ± 0.8
River water (0.56 km downstream from the effluent outlet)	36°14'56.0"N 137°57'04.7"E	Temperature	11℃	9℃	11℃	17℃	19℃	16℃	13.8 ± 3.7
		pH	7.4	7.2	7.8	6	6.8	6	6.9 ± 0.7

TABLE S2. Minimum inhibitory concentrations (MICs) of 25 antimicrobials against 75 CPOs in this study

Bacterial species	Strain	Sequence types	Carbapenemases	Others	MIC (μg/mL)																								
					Ampicillin	Piperacillin	SAM	TZP	Cefazolin	Cefotiam	Ceftazidime	Cefpodoxime	Ceftriaxone	Cefepime	Cefmetazole	Flomoxef	Aztreonam	Imipenem	Meropenem	Faropenem	Ertapenem	Gentamicin	Amikacin	Minocycline	Levofloxacin	Fosfomycin	SXT	Colistin	Tigecycline
Aeromonas allosaccharophila	10CC9	1180	GES-24	TEM-1B	>16	>64	>16-8	64-4	>16	>4	4	>4	16	8	8	16	≤2	2	4	>64	16	>8	16	≤2	1	≤32	≤9.5-0.5	8	0.5
Aeromonas caviae	4CC9	1057	GES-5		>16	64	>16-8	32-4	>16	>4	8	>4	8	≤2	>32	>16	≤2	0.5	0.5	32	4	≤2	≤8	≤2	2	≤32	38-2	≤0.25	0.5
	6BC8	1178	GES-5	MOX	>16	>64	>16-8	>64-4	>16	>4	16	>4	32	>16	>32	>16	16	0.5	1	32	16	≤2	≤8	≤2	0.5	≤32	≤9.5-0.5	1	0.5
	6CC6	1178	GES-5	MOX	>16	64	>16-8	64-4	>16	>4	8	>4	16	>16	>32	>16	8	2	4	16	8	≤2	≤8	≤2	1	≤32	>38-2	0.5	2
	10BC8	1059	GES-5	MOX+VEB-1	>16	32	>16-8	8-4	>16	>4	>16	>4	>32	8	>32	>16	>16	0.5	0.5	16	2	≤2	≤8	≤2	2	≤32	>38-2	≤0.25	1
	2CC7	1059	GES-5	MOX+VEB-1	>16	>64	>16-8	>64-4	>16	>4	>16	>4	>32	>16	>32	>16	>16	4	4	32	32	≤2	≤8	≤2	4	≤32	>38-2	0.5	2
	4CC12	1059	GES-5	MOX+VEB-1	>16	64	>16-8	32-4	>16	>4	>16	>4	>32	>16	>32	>16	>16	4	4	16	16	≤2	≤8	≤2	4	≤32	>38-2	≤0.25	≤0.25
	10CC10	1176	GES-5	MOX+VEB-1	>16	64	>16-8	16-4	>16	>4	>16	>4	>32	>16	>32	>16	>16	1	2	16	4	≤2	≤8	≤2	1	≤32	>38-2	≤0.25	0.5
	6BC6	1199	GES-24		>16	32	>16-8	≤4-4	>16	>4	4	4	4	≤2	>32	>16	≤2	≤0.25	0.5	16	8	4	≤8	≤2	≤0.12	≤32	38-2	1	2
	10BC6	1179	GES-24		>16	>64	>16-8	64-4	>16	>4	16	>4	32	≤2	>32	>16	≤2	0.5	0.25	32	2	≤2	≤8	≤2	1	≤32	>38-2	1	8
	6CC5	1062	GES-24, IMP-1		>16	>64	>16-8	32-4	>16	>4	>16	>4	>32	>16	>32	>16	≤2	4	>8	>64	32	8	≤8	≤2	2	128	>38-2	0.5	1
	2BC3	1056	GES-48		>16	16	>16-8	8-4	>16	>4	4	>4	16	≤2	>32	>16	≤2	≤0.25	0.25	64	2	≤2	≤8	≤2	0.5	≤32	≤9.5-0.5	1	0.5
	4BC2	1056	GES-48		>16	>64	>16-8	32-4	>16	>4	16	>4	>32	≤2	>32	>16	≤2	1	0.25	32	1	≤2	≤8	≤2	0.25	≤32	≤9.5-0.5	0.5	0.5
	6BC4	1056	GES-48		>16	>64	>16-8	16-4	>16	>4	8	>4	32	≤2	>32	>16	≤2	0.5	0.5	32	8	≤2	≤8	≤2	0.5	64	≤9.5-0.5	0.5	0.5
	8BC3	1056	GES-48		>16	64	>16-8	16-4	>16	>4	4	>4	16	≤2	>32	>16	≤2	0.5	0.5	16	1	≤2	≤8	≤2	1	≤32	38-2	4	2
	10BC4	1056	GES-48		>16	64	>16-8	16-4	>16	>4	8	>4	32	≤2	>32	>16	≤2	0.5	0.5	32	16	≤2	≤8	≤2	1	≤32	>38-2	4	4
	2CC8	1056	GES-48		>16	16	>16-8	16-4	>16	>4	8	>4	16	≤2	>32	>16	≤2	≤0.25	0.5	32	2	≤2	≤8	≤2	1	64	19-1	1	1
	4CC5	1056	GES-48		>16	16	>16-8	16-4	>16	>4	4	>4	16	≤2	>32	>16	≤2	0.5	0.5	16	2	≤2	≤8	≤2	1	≤32	38-2	2	1
	6CC12	1056	GES-48		>16	>64	>16-8	32-4	>16	>4	4	>4	32	≤2	>32	>16	≤2	0.5	0.5	32	2	≤2	≤8	≤2	0.5	>128	≤9.5-0.5	1	0.5
	8CC4	1182	GES-48		>16	>64	>16-8	32-4	>16	>4	>16	>4	>32	≤2	>32	>16	4	≤0.25	0.25	16	4	≤2	16	≤2	1	≤32	38-2	0.5	1
	10CC8	1056	GES-48		>16	32	>16-8	16-4	>16	>4	4	>4	32	≤2	>32	>16	≤2	0.5	0.5	32	1	≤2	≤8	≤2	0.5	≤32	≤9.5-0.5	0.5	2
	6BC7	1061	IMP-10		>16	8	>16-8	≤4-4	>16	>4	>16	>4	>32	>16	>32	>16	≤2	2	>8	>64	>64	≤2	≤8	4	2	≤32	>38-2	0.5	4
	12BC1	1015	GES-5		>16	32	>16-8	32-4	>16	>4	8	>4	16	≤2	>32	>16	≤2	1	2	32	4	≤2	≤8	≤2	0.5	≤32	≤9.5-0.5	0.5	2
	8BC4	1015	GES-5		>16	>64	>16-8	64-4	>16	>4	16	>4	32	≤2	>32	>16	≤2	1	4	16	8	≤2	≤8	4	1	≤32	≤9.5-0.5	≤0.25	8
	10BC1	1015	GES-5		>16	>64	>16-8	64-4	>16	>4	>16	>4	32	≤2	>32	>16	≤2	1	4	64	8	≤2	≤8	≤2	1	≤32	≤9.5-0.5	≤2	4
	6CC8	1182	GES-6		>16	>64	>16-8	16-4	>16	>4	>16	>4	32	≤2	>32	>16	≤2	2	2	32	32	≤2	≤8	≤2	0.5	≤32	≤9.5-0.5	0.5	≤0.25
Aeromonas dhakensis	10CC4	518	GES-24		>16	16	>16-8	8-4	>16	>4	4	>4	>32	≤2	>32	>16	8	4	4	>64	64	≤2	32	4	>4	≤32	>38-2	>64	4
Aeromonas hydrophila subsp. *hydrophila*	8CC6	1177	GES-24		>16	>64	>16-8	32-4	>16	>4	16	>4	32	4	>32	>16	≤2	>8	>8	>64	>64	≤2	≤8	4	2	≤32	>38-2	8	4
	8CC5	860	GES-24, IMP-1		>16	>64	>16-8	>64-4	>16	>4	>16	>4	>32	>16	>32	>16	4	4	>8	64	>64	≤2	≤8	≤2	4	≤32	>38-2	8	4
	6CC13	1181	GES-5		>16	>64	>16-8	64-4	>16	>4	8	>4	8	>16	>32	>16	≤2	>8	8	>64	>64	≤2	≤8	≤2	2	>128	>38-2	>64	2
Aeromonas taiwanensis	2BC2	1055	GES-5		>16	32	>16-8	16-4	>16	>4	16	>4	16	≤2	32	>16	≤2	≤0.25	≤0.12	64	2	≤2	≤8	≤2	0.25	≤32	≤9.5-0.5	1	0.5
	4BC3	1055	GES-5		>16	>64	>16-8	64-4	>16	>4	16	>4	32	≤2	>32	>16	4	2	2	>64	8	≤2	≤8	≤2	0.5	≤32	≤9.5-0.5	0.5	1
Aeromonas veronii bv. *veronii*	12BC2	1054	GES-5		>16	32	>16-8	≤4-4	>16	>4	2	4	2	≤2	16	>16	4	4	4	>64	4	≤2	≤8	≤2	2	≤32	>38-2	0.5	1
	2BC1	1054	GES-5		>16	16	>16-8	≤4-4	>16	>4	2	2	2	≤2	16	16	≤2	2	2	>64	8	≤2	≤8	≤2	4	≤32	>38-2	2	2
	2CC6	1058	GES-24		>16	64	>16-8	8-4	>16	>4	4	>4	8	≤2	32	>16	≤2	2	4	>64	4	4	≤8	≤2	0.5	64	38-2	2	0.5
	6BC9	1060	GES-49		>16	>64	>16-8	>64-4	>16	>4	>16	>4	16	≤2	>32	>16	≤2	2	1	>64	32	8	≤8	4	1	≤32	>38-2	1	4
Kluyvera cryocrescens	6BC3	-	GES-6		>16	64	>16-8	16-4	>16	>4	16	4	4	≤2	>32	8	≤2	≤0.25	0.25	32	2	≤2	≤8	4	0.5	>128	≤9.5-0.5	0.5	1
Citrobacter braakii	8BC2	110	GES-5		>16	16	>16-8	8-4	>16	>4	2	>4	2	≤2	>32	16	≤2	≤0.25	≤0.12	32	1	≤2	≤8	4	2	128	≤9.5-0.5	1	1
Citrobacter freundii	4CC11	22	GES-24	CTX-M-3+TEM-1B	>16	>64	>16-8	>64-4	>16	>4	>16	>4	>32	>16	>32	>16	16	8	1	32	1	>8	>32	>8	>4	≤32	>38-2	≤0.25	1
	2CC2	116	IMP-1	CTX-M-3+TEM-1B	>16	>64	>16-8	16-4	>16	>4	>16	>4	>32	>16	>32	>16	>16	4	8	>64	32	>8	≤8	4	1	≤32	≤9.5-0.5	4	0.5
	12CC1	116	IMP-1	CTX-M-3+TEM-1B	>16	>64	>16-8	8-4	>16	>4	>16	>4	>32	>16	>32	>16	16	4	8	>64	32	>8	≤8	>8	2	≤32	≤9.5-0.5	1	0.5
Citrobacter portucalensis	10CC7	166	GES-51		>16	64	>16-8	16-4	>16	>4	4	>4	2	≤2	>32	>16	≤2	4	4	32	32	≤2	≤8	≤2	0.5	≤32	≤9.5-0.5	≤0.25	0.5
Enterobacter cloacae subsp. cloacae	8BC1	1821	GES-4		>16	32	>16-8	≤4-4	>16	>4	8	4	2	≤2	>32	8	≤2	≤0.25	≤0.12	16	1	≤2	≤8	≤2	0.5	≤32	≤9.5-0.5	2	2
	10BC2	1821	GES-4		>16	32	>16-8	8-4	>16	>4	16	4	2	≤2	>32	16	≤2	≤0.25	≤0.12	16	≤0.5	≤2	≤8	≤2	1	≤32	≤9.5-0.5	1	1
Enterobacter kobei	6CC1	1822	GES-4		>16	16	16-8	≤4-4	>16	>4	8	4	2	≤2	>32	>16	≤2	8	4	32	8	≤2	≤8	4	1	128	≤9.5-0.5	32	2
	6BC5	520	GES-54		>16	32	>16-8	16-4	>16	>4	16	4	2	≤2	>32	16	≤2	≤0.25	0.5	64	2	≤2	≤8	8	1	128	19-1	1	1
Enterobacter roggenkampii	4CC4	1059	GES-4		>16	32	>16-8	64-4	>16	>4	>16	>4	2	≤2	>32	>16	≤2	2	2	>64	2	≤2	≤8	4	1	≤32	≤9.5-0.5	≤0.25	0.5
	6BC1	433	GES-24	mcr-9	>16	>64	>16-8	>64-4	>16	>4	>16	>4	>32	≤2	>32	>16	>16	>8	>8	>64	>64	≤2	32	4	2	≤32	38-2	32	1
Klebsiella michiganensis	12CC2	95	GES-4		>16	>64	>16-8	64-4	>16	>4	>16	>4	4	≤2	>32	>16	≤2	>8	>8	>64	32	≤2	≤8	4	≤0.12	≤32	19-1	0.5	1
	4CC2	95	GES-4		>16	>64	>16-8	64-4	>16	>4	>16	>4	4	≤2	>32	>16	≤2	8	>8	>64	32	≤2	≤8	≤2	≤0.12	64	≤9.5-0.5	≤0.25	1
	6CC2	95	GES-4		>16	64	>16-8	32-4	>16	>4	>16	>4	4	≤2	>32	>16	≤2	8	>8	64	64	≤2	≤8	4	≤0.12	≤32	≤9.5-0.5	≤0.25	1
	8CC1	95	GES-4		>16	>64	>16-8	32-4	>16	>4	>16	>4	8	≤2	>32	>16	≤2	>8	8	>64	32	≤2	≤8	4	0.25	≤32	19-1	≤0.25	2
	2CC1	135	GES-47		>16	>64	>16-8	>64-4	>16	>4	>16	>4	4	4	>32	>16	≤2	2	4	>64	4	≤2	≤8	≤2	0.5	≤32	≤9.5-0.5	1	0.5
	2BC4	210	GES-50		>16	32	>16-8	16-4	>16	4	2	≤1	≤1	≤2	16	4	≤2	1	0.25	8	1	≤2	≤8	≤2	0.25	≤32	≤9.5-0.5	1	0.5
	10BC5	210	GES-50		>16	32	>16-8	8-4	>16	4	2	≤1	≤1	≤2	16	>16	≤2	0.5	0.25	64	8	≤2	≤8	≤2	0.5	≤32	≤9.5-0.5	≤0.25	2
Klebsiella pneumoniae subsp. *pneumoniae*	6BC2	2791	GES-5		>16	>64	>16-8	32-4	>16	>4	4	4	2	≤2	>32	>16	≤2	2	2	>64	32	≤2	≤8	4	0.5	≤32	≤9.5-0.5	1	4
	4CC3	2791	GES-5		>16	>64	>16-8	32-4	>16	>4	4	4	2	≤2	>32	>16	≤2	1	2	>64	4	≤2	≤8	4	0.5	≤32	≤9.5-0.5	≤0.25	2
	6CC4	2791	GES-5		>16	64	>16-8	64-4	>16	>4	8	>4	2	≤2	>32	>16	≤2	2	4	>64	32	≤2	≤8	4	0.5	>128	≤9.5-0.5	≤0.25	4
	10CC2	2791	GES-5		>16	32	>16-8	32-4	>16	>4	4	4	2	≤2	>32	>16	≤2	1	2	>64	4	≤2	≤8	4	0.5	≤32	≤9.5-0.5	≤0.25	4
	10CC1	76	GES-48		>16	32	>16-8	16-4	>16	>4	2	2	≤1	≤2	>32	8	≤2	2	1	64	2	≤2	≤8	4	0.25	≤32	≤9.5-0.5	≤0.25	4
Klebsiella quasipneumoniae subsp. *similipneumoniae*	6CC9	6101	GES-4		>16	>64	>16-8	32-4	>16	>4	>16	2	4	≤2	>32	8	≤2	0.5	0.5	>64	2	≤2	≤8	8	0.5	≤32	≤9.5-0.5	0.5	2
	4CC10	1822	GES-5		>16	>64	>16-8	>64-4	>16	>4	16	2	4	≤2	>32	>16	≤2	0.5	0.5	32	1	≤2	≤8	≤2	1	64	≤9.5-0.5	≤0.25	0.5
	6CC7	1822	GES-5		>16	>64	>16-8	64-4	>16	>4	16	≤1	2	≤2	>32	8	≤2	0.5	0.5	>64	8	≤2	≤8	≤2	1	>128	≤9.5-0.5	0.5	0.5
	10CC6	1822	GES-5		>16	64	>16-8	64-4	>16	>4	16	≤1	2	≤2	32	8	≤2	1	0.5	32	1	≤2	≤8	≤2	1	>128	≤9.5-0.5	1	1
	8CC2	6102	GES-48		>16	32	>16-8	32-4	>16	>4	2	4	2	≤2	>32	>16	≤2	8	8	>64	32	4	≤8	>8	4	128	≤9.5-0.5	0.5	8
Raoultella ornithinolytica	4CC7	-	GES-4		>16	>64	>16-8	16-4	>16	>4	16	2	2	≤2	>32	≤2	≤2	2	0.5	>64	1	≤2	16	≤2	0.5	128	>38-2	≤0.25	0.5
	6CC3	-	GES-4		>16	32	>16-8	16-4	>16	>4	>16	4	2	≤2	>32	>16	≤2	4	4	>64	16	≤2	≤8	≤2	1	>128	≤9.5-0.5	≤0.25	≤0.25
	8CC3	-	GES-4		>16	64	>16-8	16-4	>16	>4	16	4	2	≤2	32	8	≤2	0.5	0.5	>64	4	≤2	≤8	4	0.5	64	≤9.5-0.5	≤0.25	2
	10CC5	-	GES-4		>16	32	>16-8	16-4	>16	>4	>16	4	2	≤2	>32	4	≤2	1	1	>64	2	≤2	≤8	≤2	0.5	128	≤9.5-0.5	≤0.25	1
	2CC4	-	GES-5		>16	>64	>16-8	64-4	>16	>4	8	4	2	≤2	>32	16	≤2	4	2	>64	4	≤2	≤8	4	1	>128	≤9.5-0.5	2	1
	6CC10	-	GES-5		>16	>64	>16-8	64-4	>16	>4	8	4	≤1	≤2	>32	8	≤2	2	1	>64	16	≤2	≤8	4	2	128	≤9.5-0.5	0.5	4
	4BC1	-	GES-24		>16	8	>16-8	64-4	>16	>4	2	2	≤1	≤2	32	≤2	≤2	1	1	>64	1	≤2	≤8	8	≤0.12	≤32	≤9.5-0.5	≤0.25	1
Raoultella planticola	12CC3	-	GES-5		>16	>64	>16-8	64-4	>16	>4	8	>4	32	≤2	>32	>16	≤2	4	4	>64	8	≤2	≤8	>8	>4	64	>38-2	≤0.25	1
	2CC3	-	GES-5		>16	64	>16-8	32-4	>16	>4	4	4	2	≤2	>32	>16	≤2	4	4	>64	32	≤2	≤8	4	0.25	≤32	≤9.5-0.5	4	1
	4CC1	-	GES-5		>16	>64	>16-8	64-4	>16	>4	4	4	2	≤2	>32	>16	≤2	8	4	>64	16	≤2	≤8	4	0.25	128	≤9.5-0.5	≤0.25	2

SAM, ampicillin-sulbactam; TZP, piperacillin-tazobactam; SXT, sulfamethoxazole-trimethoprim.

The MIC values interpretation into resistant (pink), intermediate (beige), and susceptible (light blue) is shown according to the Clinical & Laboratory Standards Institute (CLSI) guidelines. In case of no interpretive criteria are available, only MIC values are indicated.
